# *In vivo* modulation of endothelial polarization by Apelin receptor signalling

**DOI:** 10.1038/ncomms11805

**Published:** 2016-06-01

**Authors:** Hyouk-Bum Kwon, Shengpeng Wang, Christian S. M. Helker, S. Javad Rasouli, Hans-Martin Maischein, Stefan Offermanns, Wiebke Herzog, Didier Y. R. Stainier

**Affiliations:** 1 Department of Developmental Genetics, Max Planck Institute for Heart and Lung Research, Ludwigstrasse 43, 61231 Bad Nauheim, Germany; 2 Department of Pharmacology, Max Planck Institute for Heart and Lung Research, Ludwigstraße 43, 61231 Bad Nauheim, Germany; 3 University of Münster, Schlossplatz 2, 48149 Münster, Germany; 4 Max Planck Institute for Molecular Biomedicine, Röntgenstrasse 20, 48149 Münster, Germany

## Abstract

Endothelial cells (ECs) respond to shear stress by aligning in the direction of flow. However, how ECs respond to flow in complex *in vivo* environments is less clear. Here we describe an endothelial-specific transgenic zebrafish line, whereby the Golgi apparatus is labelled to allow for *in vivo* analysis of endothelial polarization. We find that most ECs polarize within 4.5 h after the onset of vigorous blood flow and, by manipulating cardiac function, observe that flow-induced EC polarization is a dynamic and reversible process. Based on its role in EC migration, we analyse the role of Apelin signalling in EC polarization and find that it is critical for this process. Knocking down Apelin receptor function in human primary ECs also affects their polarization. Our study provides new tools to analyse the mechanisms of EC polarization *in vivo* and reveals an important role in this process for a signalling pathway implicated in cardiovascular disease.

## Abstract (web summary)

Endothelial cells align in the direction of flow in response to shear stress. Here the authors describe a zebrafish model for visualization of endothelial polarization and demonstrate that endothelial cell alignment depends on blood flow and Apelin signalling.

## Graphical abstract

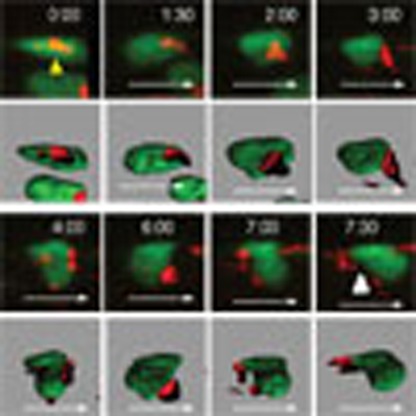

Vascular endothelial cells (ECs) form a continuous cell layer that lines blood vessels. ECs are constantly subjected to a variety of shear stresses from the flow of blood and display profound morphological adaptations to their environmental conditions. These morphological adaptations include planar cell polarization, cell elongation and alignment of microtubules in the direction of blood flow^1^,^2^. Recent studies have shown that defects in ECs' adaption to blood flow are highly correlated with cardiovascular diseases including atherosclerosis^3^,^4^. The alignment of ECs in the direction of flow requires the sensing of mechanical shear stress and the conversion of these stimuli to biochemical signals that mediate cytoskeletal remodelling. A number of mechanosensitive molecules have been identified including mechanically gated channels^5^,^6^,^7^,^8^, mechanosensitive receptors^9^,^10^,^11^, G-protein-coupled receptors (GPCRs)^12^,^13^,^14^,^15^,^16^, G-proteins^16^,^17^,^18^,^19^, mechanosensitive enzymes^20^ and cilia^21^. The observation that certain G-proteins are activated within seconds following mechanical perturbation by shear stress suggests that GPCRs are involved in mediating mechanochemical signal transduction in ECs.

Here we generated a new zebrafish transgenic line in which the Golgi apparatus is labelled with a fluorescent protein. Using this line in combination with an endothelial nuclear line allows one to follow EC polarization in real time. We analysed EC polarization during development and investigated the role of blood flow in this process. We further found that the Apelin receptor (Aplnr), a GPCR involved in EC migration, also regulates EC polarization.

## Result

### EC polarization during development

To investigate the dynamics of endothelial polarization by blood flow *in vivo*, we generated a new zebrafish transgenic line that labels the Golgi apparatus specifically in ECs^22^. We fused the complementary DNA of human β-1, 4-galactosyltransferase1 (B4GALT1), which is mainly localized to the *trans* Golgi network^23^, to mCherry and expressed it under the control of the *fli1a* endothelial promoter^24^. To validate this transgenic line, we performed confocal imaging during vascular development of *Tg(kdrl:NLS-EGFP);Tg(fli1a:B4GALT1-mCherry)* embryos, which express nuclear enhanced green fluorescent protein (EGFP) and Golgi-specific mCherry specifically in ECs. We found that during EC migration the Golgi was localized at the leading edge of cells (Supplementary Fig. 1a,b and Supplementary Movie 1). However, when ECs stopped migrating, the Golgi quickly moved to a middle position relative to the nucleus (Supplementary Fig. 1b,d). In addition, the Golgi localized to the luminal side of ECs during lumen formation (Supplementary Fig. 1c,c′). These data indicate that this reporter line allows real-time monitoring of EC polarity *in vivo* and can be used to track changes in EC polarization.

**Figure 1:**
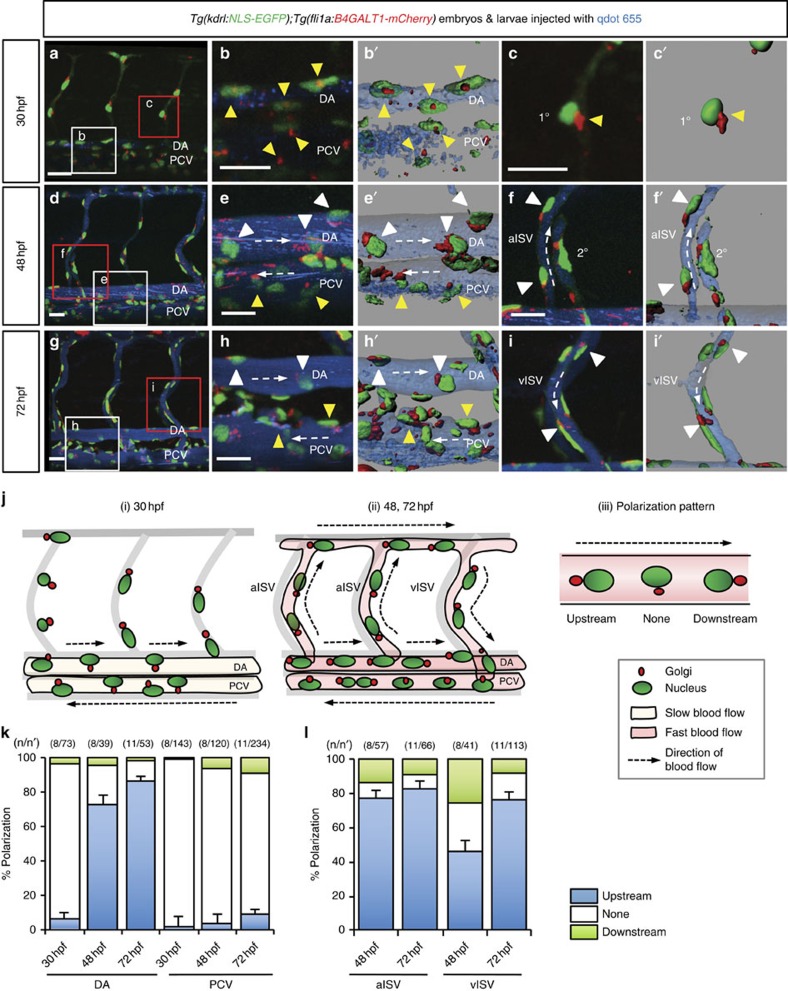
EC polarization during development. (**a**–**i'**) Three-dimensional-rendered confocal stack images of the trunk region of 30, 48 and 72 h.p.f. *Tg(kdrl:NLS-EGFP);Tg(fli1a:B4GALT1-mCherry)* animals injected intravascularly with qdot 655. The white and red boxes in the left panels (**a**,**d**,**g**) are enlarged in the middle (**b**,**e**,**h**) and right (**c**,**f**,**i**) panels, respectively. (**b'**,**c'**,**e'**,**f'**,**h'**,**i'**) Surface-rendered images of boxed regions. White dashed arrows indicate the direction of blood flow. White arrowheads point to polarized ECs and yellow arrowheads point to non-polarized ECs. (**j**) Schematic representation of EC polarization during development in the trunk region (i and ii) and illustration of the various polarization patterns (iii). (**k**,**l**) Quantification of EC polarization in the DA, PCV (**k**) and ISVs (**l**). The numbers of larvae (n) and ECs (n′) are indicated above the graph. Anterior to the left, dorsal to the top. Scale bars, 20 μm. aISV, arterial intersegmental vessel; DA, dorsal aorta; PCV, posterior cardinal vein; vISV, venous intersegmental vessel; 1°, primary sprouts; 2°, secondary sprouts. Error bars, s.e.m.

We next examined *Tg(kdrl:NLS-EGFP);Tg(fli1a:B4GALT1-mCherry)* embryos to determine whether and when ECs become polarized in response to blood flow during development. In accordance with a previous report^25^, we classify ECs as polarized when the Golgi apparatus lies upstream of the nucleus with respect to the direction of flow (Fig. 1j(iii)). At 30 hours post fertilization (h.p.f.), when blood circulation has just started (Fig. 1a–b′), ECs in the dorsal aorta (DA) and the posterior cardinal vein (PCV) remained unpolarized. However, the ECs in the DA (Fig. 1d–e′) and the intersegmental vessels (ISVs) (Fig. 1f,f′) became polarized between 48 and 72 h.p.f., a time when vigorous blood flow was established. Consistent with observations from confocal images, the percentage of polarized ECs in the DA (6.3% at 30 h.p.f., 72.9% at 48 h.p.f. and 86.5% at 72 h.p.f.) and ISVs (arterial ISVs: 77.2% at 48 h.p.f. and 80.3% at 72 h.p.f.; venous ISVs (vISVs): 43.9% at 48 h.p.f. and 76.1% at 72 h.p.f.) significantly increased as blood circulation became more vigorous (Fig. 1k,l). In contrast, most ECs in the PCV (98.2% at 30 h.p.f., 96.4% at 48 h.p.f. and 91.4% at 72 h.p.f.) failed to polarize during this time (Fig. 1k). To better understand the EC polarization differences in various vascular beds, we examined ECs in several different regions, that is, the brain, eye, pharyngeal arch, common cardinal vein (CCV) and caudal vein plexus^26^. ECs in the brain, eyes and pharyngeal arch were mostly polarized (white arrowheads), whereas those in the CCV and caudal vein plexus were not polarized as observed in the PCV (yellow arrowheads) (Supplementary Figs 2a–k and 3g,h). Interestingly, venous ECs were usually less polarized than arterial ECs. These data reveal that there is a clear heterogeneity between vascular beds in terms of EC polarization and especially between arteries and veins.

### EC polarization by blood flow

**Figure 2:**
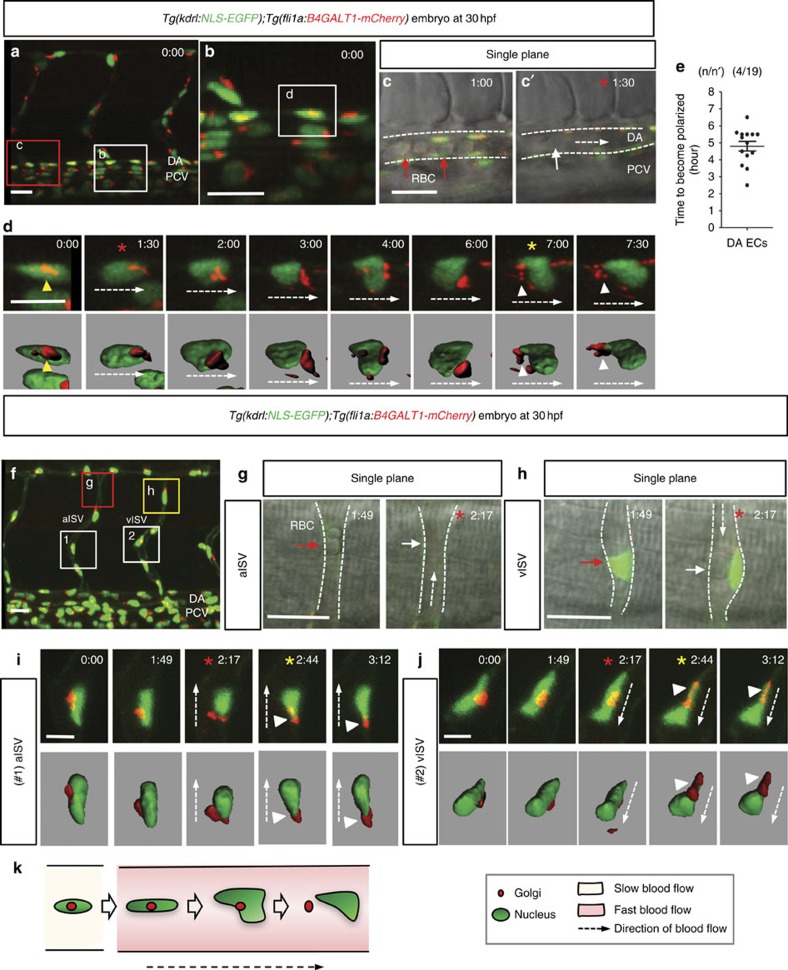
EC polarization by blood flow. (**a**–**d**) Three-dimensional-rendered confocal stack images of the trunk region of 30 h.p.f. *Tg(kdrl:NLS-EGFP);Tg(fli1a:B4GALT1-mCherry)* embryos. White box in **a** is enlarged in **b** and single plane images of red boxed region in **a** is enlarged in **c** and **c'**. (**c**,**c'**) At *t*=60 min, RBCs (red arrows) in the DA are mainly stationary, whereas by 90 min they are moving too fast to be clearly distinct (white arrow). Red asterisks indicate the time when vigorous circulation starts. (**d**) Confocal time-lapse images of EC in the white box in **b**. Surface-rendered images are displayed below. Yellow asterisk indicates the time when the EC is polarized. Time (hours:minutes) is shown in the top right corner of the images. (**e**) Quantification of time required for DA ECs to become polarized starting at 30 h.p.f. and at 28.5 °C. Numbers of larvae (**n**) and ECs (**n'**) indicated above the graph. Error bars represent s.e.m. (**f**–**j**) Three-dimensional-rendered confocal stack images of the trunk region of 30 h.p.f. *Tg(kdrl:NLS-EGFP);Tg(fli1a:B4GALT1-mCherry)* embryos. (**g**,**h**) Red and yellow boxes in **f** is enlarged in **g** and **h**. White dashed arrows indicate direction of blood flow. White dashed lines indicate ISV boundaries. (**g**) Red arrow points to stationary RBCs in the SeA; white arrow denotes apparent absence of RBCs. (**h**) At *t*=1:49, lumen formation does not appear to be completed (red arrow), whereas at *t*=2:17, a lumen appears to be formed (white arrow). (**i**,**j**) Time-lapse images of ECs marked with numbers (1 (**i**) and 2 (**j**)) in **f**. White dashed arrows indicate direction of blood flow. White arrowheads point to polarized Golgi apparatus. Red asterisks indicate the time when vigorous circulation starts. Yellow asterisks indicate the time when the EC is polarized. Time (hours:minutes) is shown in the top right corner of the images. (**k**) Schematic model showing the process of EC polarization by blood flow. Anterior to the left, dorsal to the top. Scale bars, 20 μm (**a**–**c**,**f**–**h**), 7 μm (**d**,**i**,**j**). aISV, arterial intersegmental vessel; DA, dorsal aorta; PCV, posterior cardinal vein; vISV, venous intersegmental vessel. Error bars, s.e.m.

To better understand the dynamics of EC polarization by flow, we performed time-lapse confocal imaging of ECs starting at 30 h.p.f., that is, shortly after the onset of blood flow (Fig. 2a–d). Interestingly, the Golgi apparatus in DA ECs gradually moved from a downstream to an upstream position on initiation of vigorous blood flow (Fig. 2d, Supplementary Fig. 3a–c,f(i) and Supplementary Movie 2) and it took ∼4.5 h for them to become polarized after the onset of blood flow (Fig. 2e). As blood flow gradually increases in speed after the heart starts contracting^21^, we decided to look at dividing ECs at a time when vigorous blood flow was established, to exclude this variable. EC division plane is normally oriented perpendicular to the long axis of the vessel^27^. By measuring the time required for ECs to become polarized after cell division, we observed that most resulting daughter cells became polarized in the direction of blood flow within 105 min following division (Supplementary Fig. 3d,e,f(ii) and Supplementary Movie 3). For comparison, we also analysed the correlation of EC division and polarization in the PCV by time-lapse confocal imaging. ECs within the PCV did not polarize under established blood flow, even though they divided as the DA ECs do (Supplementary Fig. 3g,h). Arterial and venous ECs have been reported to exhibit different polarization patterns *in vivo*^28^,^29^. To investigate whether arterial or venous identity plays a role in the polarization response to blood flow, we examined ECs in the arterial ISVs and vISVs, respectively. Interestingly, ECs in both vessel types polarized (Fig. 2f–j) within 30 min after the onset of blood flow, suggesting that flow-induced EC polarization may not simply be dependent on arterial or venous identity.

### EC polarization by blood flow is reversible

**Figure 3:**
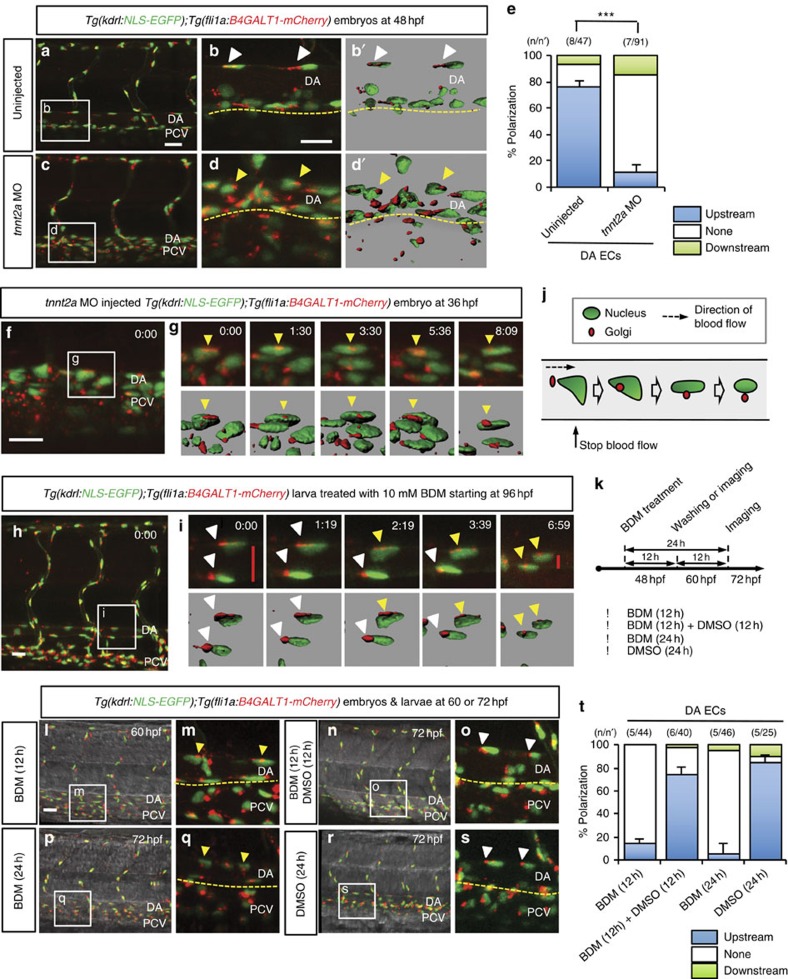
EC polarization by blood flow is reversible. (**a**–**d'**) Three-dimensional-rendered confocal stack images of 48 h.p.f. *Tg(kdrl:NLS-EGFP);Tg(fli1a:B4GALT1-mCherry)* embryos uninjected (**a**,**b**,**b'**) or injected with *tnnt2a* MO (**c**,**d**,**d'**). White boxes in left panels (**a**,**c**) enlarged in middle panels (**b**,**d**). (**b'**,**d'**) Surface-rendered images of boxed regions. White arrowheads point to polarized ECs and yellow arrowheads point to non-polarized ECs. Yellow dashed lines indicate ventral boundary of DA. (**e**) Quantification of EC polarization. Numbers of larvae (**n**) and ECs (**n'**) indicated above the graph. (**f**) Three-dimensional-rendered confocal stack images of 36 h.p.f. *Tg(kdrl:NLS-EGFP);Tg(fli1a:B4GALT1-mCherry)* embryo injected with *tnnt2a* MO. (**g**) Time-lapse confocal images of white boxed region in (**f**) surface-rendered images displayed below; yellow arrowheads point to non-polarized ECs; time (hours:minutes) shown in the top right corner of the images. (**h**) Three-dimensional-rendered confocal stack images of *Tg(kdrl:NLS-EGFP);Tg(fli1a:B4GALT1-mCherry)* larva treated with 10 mM BDM starting at 96 h.p.f. (**i**) Time-lapse confocal images of white boxed region in (**h**) surface-rendered images displayed below; white arrowheads point to polarized ECs and yellow arrowheads point to depolarized ECs; red bars indicate diameter of the DA; time (hours:minutes) shown in the top right corner of the images. (**j**) Schematic representation of EC depolarization when blood flow stops. (**k**) Schematic time table of BDM treatment of *Tg(kdrl:NLS-EGFP);Tg(fli1a:B4GALT1-mCherry)* embryos before 72 h.p.f.. (**l**–**s**) Three-dimensional-rendered confocal stack images of *Tg(kdrl:NLS-EGFP);Tg(fli1a:B4GALT1-mCherry)*embryos and larvae treated with 10 mM BDM for 12 h (**l**,**m**) or 10 mM BDM for 12 h, then dimethylsulfoxide (DMSO) for 12 h (**n**,**o**), or treated continuously with 10 mM BDM (**p**,**q**) or DMSO (**r**,**s**) for 24 h. White arrowheads point to polarized ECs and yellow arrowheads point to non-polarized ECs. (**t**) Quantification of EC polarization of 60 or 72 h.p.f. *Tg(kdrl:NLS-EGFP);Tg(fli1a:B4GALT1-mCherry* embryos and larvae treated with BDM for 12 h, BDM for 12 h then DMSO for 12 h, BDM for 24 h and DMSO for 24 h. Numbers of larvae (**n**) and ECs (**n'**) are indicated above the graph. Anterior to the left, dorsal to the top. Scale bars, 20 μm. DA, dorsal aorta; PCV, posterior cardinal vein. Error bars, s.e.m.

To further investigate the role of blood flow in EC polarization, we eliminated cardiac function by injecting morpholinos (MOs) for *tnnt2a*, a gene encoding the thin-filament contractile protein cardiac troponin T^30^, into one-cell stage *Tg(kdrl:NLS-EGFP);Tg(fli1a:B4GALT1-mCherry)* embryos. Most ECs in the DA of 48 h.p.f. *tnnt2a* morphants failed to polarize (89.1% non-polarized), as they displayed a random Golgi orientation, in contrast to ECs within the DA of uninjected embryos (76.6% polarized) (Fig. 3a–g). Likewise, when we stopped the heartbeat of 96 h.p.f. larvae with a 10 mM 2, 3-butanedione monoxime (BDM) treatment, we observed the gradual movement of the Golgi apparatus from an upstream to a downstream position within minutes after the cessation of blood flow and the ECs eventually became completely depolarized (Fig. 3h–j and Supplementary Movie 4)^31^. We observed a similar loss of polarization in the ISV ECs after stopping the blood flow (Supplementary Fig. 4a–c).

To investigate the extent of the flexibility of EC polarization, we examined ECs through a cycle of blood flow manipulation. We first stopped blood flow to depolarize the ECs and then started flow again, to analyse whether ECs were able to repolarize (Fig. 3k). We found that most ECs in the DA completely depolarized in larvae treated with BDM for 12 h (86.4%;) or 24 h (93.5%; Fig. 3p,q,t). However, 12 h after BDM removal to reinitiate blood flow, ECs repolarized to an extent (72.5%; Fig. 3n,o,t) similar to control larvae treated with dimethylsulfoxide (84.0%; Fig. 3r–t). These data indicate that EC polarization can be dynamically modulated by blood flow *in vivo*. We also investigated the role of red blood cells (RBCs) in EC polarization and depleted RBCs by injecting a *gata1* MO^32^,^33^. We found no significant differences in EC polarization between *gata1* morphants and uninjected controls at 96 h.p.f. (Supplementary Fig. 4d–g′). These data indicate that blood flow, but not the presence of RBCs, induces EC polarization.

### Aplnr signalling modulates EC polarization

**Figure 4:**
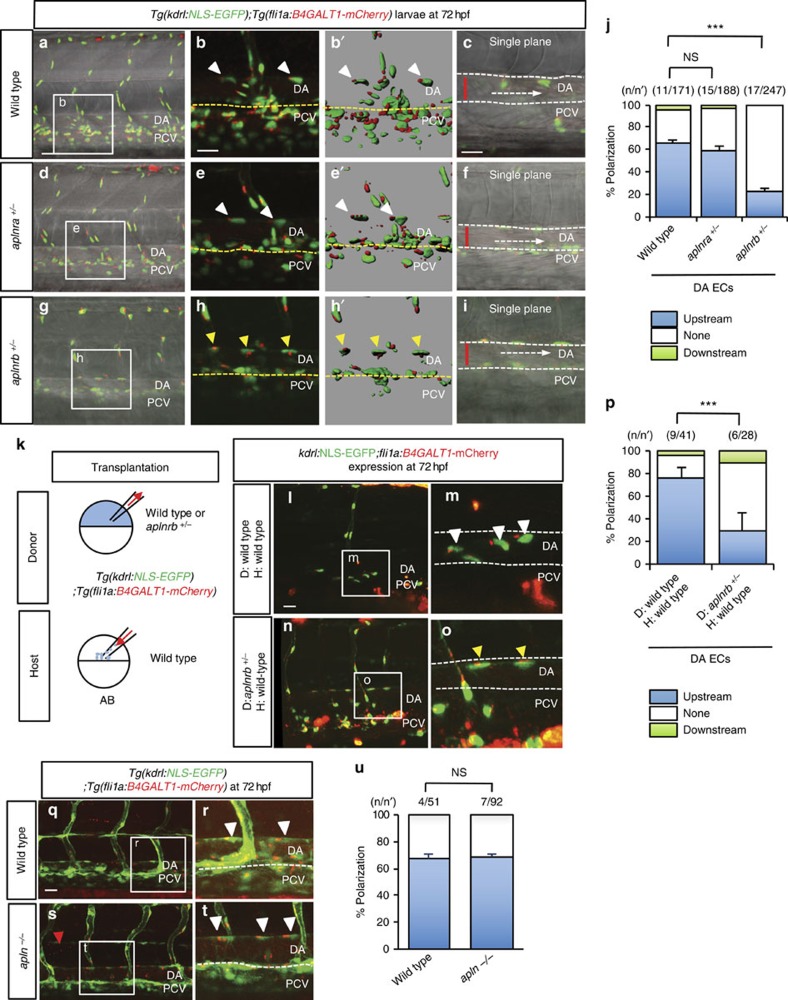
Aplnr signalling modulates EC polarization. (**a**–**i**) Confocal images of 72 h.p.f. *Tg(kdrl:NLS-EGFP);Tg(fli1a:B4GALT1-mCherry)* wild-type (**a**–**c**) *aplnra*^+/−^ (**d**–**f**) and *aplnrb*^+/−^ (**g**–**i**) larvae. Left panels: (**a**,**d**,**g**) three-dimensional (3D)-rendered confocal images of the trunk region; left centre panels: (**b**,**e**,**h**) 3D-rendered confocal images of the white boxed region in the left panels; right centre panels: (**b'**,**e'**,**h'**) 3D surface-rendered images of the centre left panels; right panels: (**c**,**f**,**i**) single plane images of the boxed region in the left panels. White arrowheads point to polarized ECs and yellow arrowheads point to non-polarized ECs. Yellow dashed lines denote the ventral boundary of the DA. White dashed lines denote the boundary of the DA in the single plane images. Red bars indicate the diameter of the DA. White dashed arrows denote the direction of blood flow. (**j**) Quantitative analysis of EC polarization in 72 h.p.f. wild type, *aplnra*^+/−^ and *aplnrb*^+/−^ larvae. The numbers of larvae (**n**) and ECs (**n'**) are indicated above the graph. ***P<0.05. (**k**) Design of the transplantation experiments: *Tg(kdrl:NLS-EGFP);Tg(fli1a:B4GALT1-mCherry)* wild type or *aplnrb*^+/−^ cells were transplanted into wild type AB hosts and the transgenic ECs in the DA scored for polarization at 72 h.p.f.. (**l**–**o**) Three-dimensional-rendered confocal images of 72 h.p.f. *Tg(kdrl:NLS-EGFP);Tg(fli1a:B4GALT1-mCherry)* wild-type hosts with wild-type (**l**,**m**) or *aplnrb*^+/−^ (**n**–**o**) transplanted cells. (**p**) Quantitative analysis of EC polarization of wild type or *aplnrb*^+/−^ cells transplanted into wild-type hosts. The numbers of larvae (**n**) and ECs (**n'**) are indicated above the graph. (**q**–**t**) Confocal images of 72 h.p.f. *Tg(kdrl:NLS-EGFP);Tg(fli1a:B4GALT1-mCherry)* wild type (**q**,**r**,**u**) and *apln*^*−/−*^ (**s**–**u**) larvae. The white boxes in the left panels (**q**,**s**) are enlarged in the right panels (**r**,**t**). (**u**) Quantitative analysis of EC polarization in 72 h.p.f. wild type and *apln*^*−/−*^ larvae. The numbers of larvae (**n**) and ECs (**n'**) are indicated above the graph. Anterior to the left, dorsal to the top. Scale bars, 20 μm. D, donor; DA, dorsal aorta; H, host; PCV, posterior cardinal vein. Error bars, s.e.m.

Based on our *in vivo* observations, EC migration (Supplementary Fig. 1a,b,d) and blood flow (Fig. 2d,i–k) induce the same polarization response. Thus, we decided to investigate the role of signalling pathways known to regulate EC migration during EC polarization. The Aplnr is a GPCR implicated in the migration of ECs and cardiac precursor cells^34^,^35^,^36^,^37^. In addition, *Aplnr* is strongly expressed in the cardiovascular system of mouse, frog and zebrafish, and it plays an important role in cardiovascular function^38^,^39^,^40^. In zebrafish, there are two paralogues of *aplnr*, *aplnra* and *aplnrb*, and two ligand-encoding genes, *apelin* (*apln*) and *apela*^41^,^42^. We examined the expression of these genes by *in situ* hybridization and found that *aplnra*, *aplnrb* and *apln* were highly expressed in the zebrafish vasculature during development (Supplementary Fig. 5a–c), whereas *apela* was not (Supplementary Fig. 5d), consistent with previous reports^41^. As we did not detect *apela* expression in developing blood vessels, we decided to focus on *aplnra*, *aplnrb* and *apln*. We first analysed *aplnr*, *aplnra* and *aplnrb* mutant larvae. We did not observe any obvious phenotype in *aplnra*^+/−^ or *aplnra*^*−/−*^ larvae (Supplementary Fig. 5e). As previously reported^34^,^35^, *aplnrb*^*−/−*^ animals develop pericardial oedema (red arrow, Supplementary Fig. 5e), whereas *aplnrb*^+/−^ animals exhibit no obvious phenotype (Supplementary Fig. 5e). However, analysis of Golgi localization in *aplnrb*^+/−^ larvae at 72 h.p.f. revealed defects in EC polarization (23.1%) despite vigorous blood flow (Fig. 4g–i,j and Supplementary Fig. 5f–i). In contrast, EC polarization in *aplnra*^+/−^ larvae (59.6%; Fig. 4d–f,j) appeared similar to wild-type siblings (66.7%; Fig. 4a–c,j). These data suggest that *aplnrb* modulates EC polarization. To test whether Aplnrb signalling modulates EC polarization cell autonomously, we generated mosaic embryos by cell transplantation (Fig. 4k). We observed a reduction in the polarization frequency of *aplnrb*^+/−^ ECs transplanted into wild-type larvae (14.3%; *n*/*n*'=6/28), compared with that of wild-type ECs transplanted into wild-type larvae (63.4%; *n*/*n*'=9/41) (Fig. 4l–o,p), indicating that Aplnrb signalling modulates EC polarization in a cell-autonomous manner.

To investigate whether Apelin ligands also regulate EC polarization, we first examined *apln* morphant larvae at 72 h.p.f.^38^. To identify a dose of *apln* MO that causes minimal presumed off-target effects, we injected *apln* MO into embryos from an *apln*^+/−^ incross^43^ (Supplementary Fig. 6a–f,g) and selected 4.5 ng. At this dose, *apln* morphants exhibited a complete block in parachordal vessel formation (Supplementary Fig. 6h–j) as previously reported^38^, but also minimal off-target effects, for example, pericardial oedema (Supplementary Fig. 6a–g). Interestingly, *apln* morphants showed a similar frequency of EC polarization (60.6%, Supplementary Fig. 6n–p,q) as uninjected controls (70.6%; Supplementary Fig. 6k–m,q). We further investigated whether EC polarization was dependent on Apln by evaluating *apln*^*−/−*^ larvae at 72 h.p.f. (Fig. 4q–u). *apln*^*−/−*^ larvae showed a lack of parachordal vessels similar to *apln* morphants (Fig. 4s and Supplementary Fig. 6h–j). In addition, no significant effect on in EC polarization was observed in *apln*^*−/−*^ larvae (68.5%; Fig. 4s–u) compared with wild-type siblings (67.4%; Fig. 4q,r,u). These data suggest that Aplnrb modulates EC polarization independently of Apln function. We next examined the expression of *apela* in *apln*^*−/−*^ animals, to see whether there was an upregulation of *apela* expression that might compensate for the loss of Apln function. However, *apela* expression did not appear to be significantly increased in *apln*^*−/−*^ embryos or larvae compared with wild-type siblings (Supplementary Fig. 7a,b).

In mouse, Apln has been linked to tip/stalk cell differentiation during sprouting angiogenesis and Apln is dysregulated in Dll4 loss-of-function models^44^. Loss of Apln or Aplnr causes delayed blood vessel development in the mouse retina and reduces EC proliferation during fin regeneration in zebrafish^44^. We thus counted EC numbers in ISVs of *aplnrb*^+/−^ and *apln*^*−/−*^ larvae, to determine whether cell proliferation was reduced. As previously reported, both *aplnrb*^+/−^ and *apln*^*−/−*^ larvae showed reduced EC numbers in ISVs (Supplementary Fig. 7c,d). However, and as previously mentioned, *apln*^*−/−*^ larvae do not show an endothelial polarization defect (Fig. 4q–u), suggesting that reduction in cell proliferation is not sufficient to cause a polarization defect in ECs. In addition, we investigated whether loss of Aplnrb affected arterial-venous identity or Notch signalling. We did not observe any obvious differences between wild-type and *aplnrb*^+/−^ embryos and wild-type siblings in the expression of the arterial reporter line *Tg(0.8flt1:RFP)* (Supplementary Fig. 7e–f′) or *vegfr3* in the PCV (Supplementary Fig. 7g–i). Furthermore, no differences in the expression of the Notch reporter line *Tg(EPV.TP1-Mmu.Hbb:hist2h2l-mCherry)*^45^ were observed between *aplnrb*^+/−^ embryos and wild-type siblings (Supplementary Fig. 7j–k′).

### APLNR signalling modulates the polarization of human ECs

**Figure 5:**
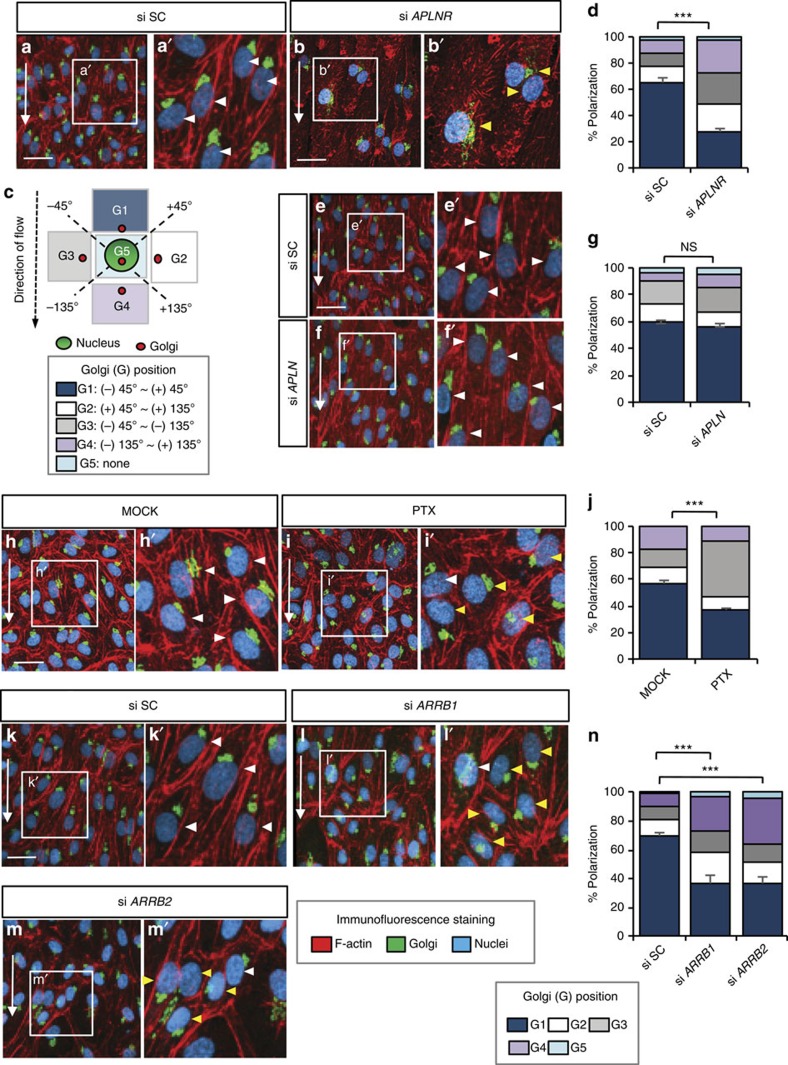
APLNR signalling modulates the polarization of human ECs. (**a**–**b'**,**e**–**f'**,**h**–**i'**,**k**–**m'**) Immunofluorescence staining of HUVECs transfected with si SC or si *APLNR* (**a**–**b'**), transfected with si SC or si *APLN* (**e**–**f'**) treated with water (MOCK) or the Gαi inhibitor PTX (100 ng ml^−1^) (**h**–**i'**) and transfected with si SC, si *ARRB1* or si *ARRB2* (**k**–**m'**) subjected to laminar flow at 12 dynes cm^−2^ for 18 h. Cells were fixed and stained with the GM130 Golgi antibody (green), Phalloidin (red) and 4,6-diamidino-2-phenylindole (DAPI, blue). White arrowheads point to polarized ECs (that is, Golgi apparatus positioned within ±45° against the direction of laminar flow (G1)). Yellow arrowheads point to depolarized ECs (tht is, Golgi positioned within+45° to +180° and +180° to −45° against the direction of laminar flow (G2–G4)). (**c**) Schematic representation showing the classification of polarization: The orientation of the Golgi apparatus relative to the nucleus was assessed to define cell polarization under laminar flow. (**d**,**g**,**j**,**n**) Quantitative analysis of polarization of HUVECs subjected to laminar flow at 12 dynes cm^−2^ for 18 h treated with si SC or si *APLNR* (**d**) transfected with si SC or si *APLN* (**g**) treated with water (MOCK) or the Gαi inhibitor PTX (100 ng ml^−1^) (**j**) transfected with si SC, si *ARRB1* or si *ARRB2* (**n**). *n*>300 cells, from at least three independent experiments. Scale bars, 20 μm. Error bars, s.e.m.

To test whether the role of Aplnr in EC polarization was conserved in humans, we investigated EC polarization *in vitro* after knocking down *APLNR* (also known as APJ and AGTRL1) in primary human ECs, human umbilical venous ECs (HUVECs), under laminar flow (Fig. 5a–b′). Interestingly, only 27.6% of *APLNR* knockdown (KD) HUVECs under laminar flow showed a polarized localization of their Golgi apparatus compared with 64.8% of control cells (white arrowheads; Fig. 5a–b′), as defined by the position of the Golgi apparatus within a 45° angle of the direction of flow (Fig. 5c). In addition, APLNR KD HUVECs showed severely disturbed cytoskeletal structures compared with control cells, which show stress fibres aligned along the direction of flow (Fig. 5a′). We observed similar defects in Golgi localization and stress fibre assembly when we knocked down *APLNR* in human arterial ECs (HUAECs) (Supplementary Fig. 8a–b′,c). These results suggest that the role of Aplnr in EC polarization is conserved in humans.

The APLNR has been reported to function in mouse cardiomyocytes as a dual receptor for Apln via G-protein αi (Gαi) and mechanical stretch via β-Arrestin (Arrb)^46^. To analyse whether the role of APLNR in laminar flow-induced EC polarization was dependent on its ligand APLN and Gαi, we knocked down APLN (Fig. 5e–g) and also inhibited Gαi signalling by treatment with the Gαi inhibitor pertussis toxin (PTX) (Fig. 5h–j). We did not find a significant effect on EC polarization or the actin cytoskeleton in *APLN* KD HUVECs (56.5%) under laminar flow, compared with controls (59.6%) (Fig. 5e–g). However, we observed a moderate decrease in EC polarization under laminar flow in PTX-treated HUVECs (36.6%) compared with controls (56.6%) (Fig. 5h–j). Given that the exposure to PTX was relatively long (>30 h due to the experimental design), it is likely to be that the effect of PTX is not specific to inhibiting APLNR downstream signalling. Next, we investigated whether laminar flow-induced EC polarization was dependent on ARRB. We found depolarized Golgi localization as well as disrupted actin cytoskeleton when both *ARRB* isoforms, *ARRB1* and *ARRB2*, were knocked down compared with controls (Fig. 5k–m′). These data suggest that EC polarization may be modulated in an APLN-independent manner, and that APLNR-dependent EC polarization may function, at least in part, via ARRB. We also analysed whether ARRB is an effector of APLNR in laminar flow-induced signalling by transfecting ARRB-GFP into HUAECs. We found that ARRB-GFP translocated to the membrane when HUAECs were exposed to laminar flow, but not after *APLNR* KD (Supplementary Fig. 8d–g′,h). These data suggest that ARRB functions downstream of APLNR in a flow-dependent signalling.

### *KLF2* regulation by APLNR and EC polarization in *klf2* mutants

Aplnr mutant mice show decreased endothelial expression of *Krüppel-like factor 2* (*Klf2*), which encodes a master regulator of vascular adaptation to blood flow^47^. Thus, we examined whether *KLF2* expression was reduced in *APLNR* KD HUVECs under laminar flow and in *aplnrb* heterozygous and homozygous mutant zebrafish embryos. We found that *KLF2* expression was reduced in *APLNR* KD HUVECs under laminar flow compared with controls (Supplementary Fig. 9a). However, there did not appear to be a dramatic difference in *klf2a* expression between wild-type and *aplnrb*^+/−^ zebrafish embryos, and although *klf2a* expression in *aplnrb*^*−/−*^ is clearly reduced compared with wild type, these animals have no blood flow that probably accounts, at least in part, for this reduction in expression (Supplementary Fig. 9b–d). To investigate whether loss of Klf2 function leads to endothelial polarization defects to a similar extent as that observed in *aplnrb*^+/−^ larvae, we examined zebrafish mutants for the two *klf2* paralogues, *klf2a* and *klf2b*. Both *klf2a* and *klf2b* single mutants appear to develop normally (Supplementary Fig. 9e) in agreement with a recent report^48^. However, we found that 50% of *klf2a*^*−/−*^ larvae (*n*=6), 83% of *klf2b*^*−/−*^ larvae (*n*=6) and 80% of *klf2a* MO-injected *klf2b*^*−/−*^ larvae showed EC polarization defects as observed in *aplnrb*^+/−^ larvae (Supplementary Fig. 9f,g–k′). We also observed that loss of *klf2* function reduced the diameter of the DA and PCV (Supplementary Fig. 9l–n′,o) and increased blood flow velocity (Supplementary Fig. 9p,q), indicating that the polarization defects are not due to decreased blood flow velocity. Altogether, these data suggest that Aplnr may modulate EC polarization and vascular remodelling through Klf2.

## Discussion

In this study, we found that EC polarization was heterogeneous between different vascular beds (Fig. 1a–i,k,l and Supplementary Fig. 2a–k). Previous studies have reported different polarization patterns in arterial and venous ECs in fixed tissue samples^28^,^29^. Consistent with these reports, we observed that ECs in the DA polarize shortly after the onset of vigorous blood flow (Fig. 2a–d), whereas ECs in the PCV do not (Supplementary Fig. 3g,h). Interestingly, some non-polarized ECs in the PCV became polarized after migrating dorsally to form the vISVs (Fig. 2f,h,j). These data suggest that ECs' ability to respond to flow is not fixed by arterial or venous identity, but dynamically adjusted according to their environment. Factors such as the velocity and pulse patterns of flow are known to influence EC polarization *in vivo* and *in vitro*^41^,^49^. Anton *et al*.^50^ and Goetz *et al*.^21^ analysed the differences in blood cell motion in the DA, PCV and ISVs of zebrafish embryos. Blood cell motion in the DA (mean velocity of ∼300 μm s^−1^; shear stress of ∼0.43 dynes cm^−2^) reflects the contractions of the heart, that is, with regular intermittent propagation of fluid and almost no flow between pulses. ISV flow is also pulsatile but with a profile significantly different from that of the DA. Its peak flow is slower than that of the DA. By contrast, blood flow in the PCV (mean velocity of ∼100 μm s^−1^; shear stress of ∼0.14 dynes cm^−2^) is relatively steady with a 57% reduction in pulse amplitude. Thus, changes in blood flow velocity and pulse patterns may affect endothelial response to flow, as ECs move from the PCV into vISVs. In addition, Baeyens *et al*.^51^ reported that Vegfr3 signalling regulates the shear stress response of ECs in different blood vessels including arteries, veins and lymphatics. However, the relative contribution of different extrinsic and intrinsic signals in ECs' response to shear stress remains to be investigated.

Using live imaging, we observed that ECs become polarized during their migration (Supplementary Fig. 1a–d) and also after exposure to flow (Fig. 2a–d,k). Aplnr is known to regulate EC migration during development^36^. Previously, Scimia *et al*.^46^ reported that in mouse cardiomyocytes, the Aplnr transduces Apln binding or mechanical stretch in different ways. The response to Apln seems to involve G-proteins, as it is PTX sensitive, whereas that induced by stretch occurs in the absence of Apln, which is PTX insensitive and G-protein independent but Arrb dependent^46^. However, whether the Aplnr acts in ECs as a dual receptor for Apln and mechanical stimuli as it does in cardiomyocytes is unclear. Our studies on *aplnr* mutants reveal that ECs in *aplnrb*^+/−^ larvae fail to polarize under blood flow (Fig. 4g–i,j). Consistently, *APLNR* depleted primary human ECs showed polarization defects under laminar flow (Fig. 5a–b′), indicating that the role of Aplnr in EC polarization is conserved. Interestingly, ECs in *apln* mutant and *apln* morphant larvae did not show a significant difference in polarization compared with controls (Fig. 4q–u and Supplementary Fig. 6k–p,q). Similarly, *APLN* KD HUVECs did not show a significant reduction in polarization compared with controls (Fig. 5e–f′). Furthermore, HUVECs deficient for *ARRBs* showed a reduction in EC polarization under laminar flow (Fig. 5k–n). Altogether, these data suggest an Apln-independent function of Aplnr in flow-induced EC polarization and one that requires at least in part Arrb function. However, even though EC polarization appeared mostly unaffected in *apln* mutants and morphants, as well as in *APLN* KD HUVECs under laminar flow, we cannot exclude the possibility of ligand dependency, for example, via Apela or an unknown ligand. Ligand dependency could still be the case if the residual levels of Apela in *apln* mutants and morphants or in *APLN* KD HUVECs are sufficient to induce EC polarity, or if another ligand, even possibly a heretofore unknown ligand, can activate Aplnr during EC polarization.

*KLF2* expression was reduced in *APLNR* KD HUVECs (Supplementary Fig. 9a) and in *aplnrb*^+/−^ or *aplnrb*^*−/−*^ zebrafish embryos (Supplementary Fig. 9b–d). Previous studies have suggested that Klf2 is essential for vascular homeostasis^52^,^53^. In this study, we observed reduced blood vessel diameter in larvae when Klf2 function was compromised (Supplementary Fig. 9l–n′,o), suggesting that Klf2 plays an important role during vascular remodelling. Furthermore, although *klf2a*^*−/−*^ and *klf2a*^*−/−*^; *klf2b*^+/−^ zebrafish larvae exhibited increased blood flow velocity (Supplementary Fig. 9q), we observed EC polarization defects in *klf2a*^*−/−*^, *klf2b*^*−/−*^ and *klf2a* MO-injected *klf2b*^*−/−*^ zebrafish larvae (Supplementary Fig. 9g–k′), indicating that EC polarization defects are not caused by reduced blood flow velocity in *klf2*-compromised zebrafish larvae.

In summary, we have developed a new tool that allows the *in vivo* visualization of the Golgi apparatus and thereby the polarization status of ECs. We observed a dynamic and reversible localization of the Golgi apparatus in ECs in response to blood flow. Interestingly, haploinsufficiency in zebrafish of the Aplnrb hampered EC polarization in the presence of normal blood flow and in a cell-autonomous manner. These results indicate that ECs process local signals to regulate their polarization through Aplnrb. Furthermore, we found this mechanism to be operating in human cells, as Aplnr KD abrogated Golgi polarization under laminar flow. Therefore, this new zebrafish transgenic line represents a valuable tool to investigate *in vivo* the pathways that modulate flow sensing and response. A better understanding of these mechanisms will probably have implications towards treating numerous pathological conditions including cardiovascular diseases.

## Methods

### Zebrafish

All zebrafish husbandry was performed under standard conditions in accordance with institutional (Max Planck Society) and national ethical and animal welfare guidelines. Embryos were staged by h.p.f. at 28.5 °C^54^. *aplnra*^*mu296*^, *aplnrb*^*mu281*^ and *apln*^mu267^ mutant lines were previously reported^36^. *klf2a*^*bns11*^ and *klf2b*^*bns12*^ were generated using TALENs. We used wild-type (AB), *Tg(kdrl:NLS-EGFP)*^*zf109*^ (ref. 55), *Tg(fli1a:B4GALT1galT-mCherry)*^*bns9*^, *Tg(-0.8flt1:RFP)*^*hu5333*^ (ref. 56) and *Tg(EPV.TP1-Mmu.Hbb:hist2h2l-mCherry)*^*s939*^ (ref. 45), *TgBAC(etv2:EGFP)*^*ci*^ (ref. 57) and *Tg(fli1a:EGFP)*^*y1*^ (ref. 58) embryos.

### Assessment of EC polarization

Polarization patterns of ECs were defined as follows: (1) upstream, if the Golgi was located on the upstream side of the nucleus; (2) none, if the Golgi was located by the middle of the nucleus; or (3) downstream, if the Golgi was located on the downstream side of the nucleus. For quantitative analyses of EC polarization, we analysed ECs of the DA, PCV and ISVs at the 5th and 6th somites at 30, 48 and 72 h.p.f. (Fig. 1k,l). To assess EC number, we counted the number of ECs located in the upper half of the DA. For quantitative analyses of EC polarization in *aplnr* heterozygous mutant larvae, we analysed ECs of the DA between, and including, the 5th and 11th somites at 72 h.p.f.

### Plasmid construction and transgenesis

The *pTol ISceI fli1a:mCherry* plasmid was constructed by modifying a pTol2 vector and inserting the *fli1a* promoter, to drive expression of the reporter protein and I-SceI meganuclease recognition sequences (5′-TAGGGATAACAGGGTAAT-3′) and *mCherry* cDNA. The *pTol ISceI fli1a:B4GALT1-mCherry* plasmid was constructed by inserting a cDNA encoding amino acids 1–60 of the human *B4GALT1* into the *pTol ISceI fli1a:mCherry* plasmid.

*Tg(fli1a:B4GALT1-mCherry)*^*bns9*^ fish were generated by injecting *pTol ISceI fli1a:B4GALT1-mCherry* (25 ng), along with *Tol2 transposase* mRNA (25 ng) into one-cell stage AB embryos. Larvae were selected at 3 days post fertilization for high expression and grown to adulthood. Germline founders were identified by specific expression of B4GALT1-mCherry in the blood vessels of their progeny.

### MO oligonucleotides and quantum dot injections

MOs (*apln* MO (4.5 ng), *tnnt2a* MO (1 ng), *gata1* MO (1ng) and *klf2a* (2ng)) were injected into one-cell stage embryos. The sequences of MOs used were as follows: *apln* MO, 5′-AACAGCCGTCACGCTCCCGACTTAC-3′)^38^; *tnnt2a* MO, 5′-CATGTTTGCTCTGATCTGACACGCA-3′ (ref. 30); *gata1* MO, 5′-CTGCAAGTGTAGTATTGAAGATGTC-3′ (ref. 32); and *klf2a* MO, 5′-gtaaaatcgttccactcaaagccat-3′ (ref. 32) (Gene Tools). One to two nanolitres of Quantum dot 655 (qdot 655) (Molecular Probes) was injected into the CCV of *Tg(kdrl:NLS-EGFP);Tg(fli1a:B4GALT1-mCherry)* embryos and larvae at 30, 48 and 72 h.p.f.

### *In vivo* imaging and image processing

Pigmentation of embryos and larvae was inhibited by 1-phenyl-2-thiourea (Sigma). The embryos were treated with 100 μg ml^−1^ tricaine (Sigma), mounted in a drop of 1.5% low melting agarose in egg water and placed onto a glass dish. Fluorescence images were obtained using a Zeiss LSM 780 confocal microscope. Three-dimensional-rendered *z*-stack images and three-dimensional surface-rendered images and movies were analysed and assembled using the IMARIS software (BITPLANE).

### Chemical treatment

Forty-eight, 60 and 72 h.p.f. *Tg(kdrl:NLS-EGFP);Tg(fli1a:B4GALT1-mCherry)* zebrafish embryos and larvae were incubated for 12 or 24 h in egg water containing 10 mM of BDM (Sigma).

### Cells and reagents

HUVECs and human umbilical artery ECs (HUAECs) were obtained from Lonza. HUVECs were cultured on collagen-I-coated dishes and supplied with the endothelial growth medium EGM-2 (Lonza). Confluent cells at passages <P3 were used for all experiments.

Pre-designed small interfering RNAs (siRNAs) against human *APLNR* (si *APLNR*), human *APLN* (si *APLN*)^46^ (Sigma-Aldrich), human *ARRB1* (si *ARRB1*) and human *ARRB2* (si *ARRB2*) (Dharmacon), as well as non-targeting control scrambled siRNA (si SC) (Invitrogen) were obtained from commercial sources.

HUVECs were transfected with siRNAs using Opti-MEM and Lipofectamine RNAiMAX (Invitrogen) according to the manufacturer's instructions. A repeated KD was performed the next day and assays were started at 72 h after the first KD. RNA isolation was performed using Qiagen RNeasy kit. Reverse transcription was done using the Transcriptor high-fidelity cDNA synthesis kit from Roche. Primers were designed with the online tool provided by Roche and quantification was performed using the LightCycler 480 Probe Master System (Roche).

For ARRB localization assays, HUAECs were transfected with predesigned siRNAs against human *APLNR* (si *APLNR*) or non-targeting control scrambled siRNA (si SC) or the pcDNA3.1 ARRB-GFP plasmid using Opti-MEM and Lipofectamine 2000 (Invitrogen) according to the manufacturer's instructions. A repeat transfection was performed the next day. To assess ARRB localization in response to laminar flow, HUAECs were grown in a μ-slide VI 0.4 flow chamber (Ibidi, Germany) until confluence. Unidirectional laminar flow was generated by a computer-controlled setup containing an air–pressure pump and a two-way switching valve (Ibidi pump system)^59^. For quantitative analysis of ARRB localization in HUAECs, we exposed cells treated with si SC or si *APLNR* to laminar flow at 20 dynes cm^−2^ for 15 min. We then analysed the plasma membrane of the GFP-positive cells.

### Immunofluorescence staining

HUVEC and HUAEC cells were fixed for 10 min with 2% paraformaldehyde (PFA), permeabilized for 10 min with 0.5% Triton X-100 in PBS, blocked for 30 min with 5% sheep serum and then probed with mouse anti-GM130 antibody (1:100 dilution, BD Bioscience), Alexa Fluor 568 Phalloidin (1:500 dilution, Molecular Probes) and 4,6-diamidino-2-phenylindole (1:1,000 dilution). Images were captured with a × 25 objective mounted on an LSM800 confocal microscope.

### Flow experiments and PTX treatment

HUVEC cells were grown in a μ-slide VI 0.4 flow chamber (Ibidi) until confluence. Unidirectional laminar flow was generated by a computer-controlled setup containing an air–pressure pump and a two-way switching valve (Ibidi pump system).

For PTX experiments, HUVEC cells were pretreated with 100 ng ml^−1^ PTX (P7208, Sigma) for 15 h then subjected to 18 h laminar flow with PTX (100 ng ml^−1^) in the flow medium.

### Whole mount *in situ* hybridization

For *in situ* hybridization, zebrafish embryos and larvae were fixed in 4% PFA overnight at 4 °C and subsequently dehydrated in methanol and stored at −20 °C until required. Before hybridization, embryos were rehydrated to PBS/0.1% Tween and then digested in 10 μg ml^−1^ Proteinase K (Roche) followed by fixation in 4% PFA in PBS/0.1% Tween. Embryos were washed in PBS/0.1% Tween, pre-incubated in hybrization buffer at 70 °C for 4 h and then incubated with Dig-labelled RNA probes in hybridization buffer at 70 °C overnight. After washing, the hybridized probes were detected with alkaline-phosphatase-conjugated, alkaline-phosphatase-labelled anti-digoxigenin antibody (11093274910, Roche, dilution 1:1,000) at 4 °C overnight and the signal was visualized with BM purple (1144207001, Roche).

Probes for *apln*, *aplnra*, *aplnrb*, *apela*, *klf2a* and *vegfr3* were amplified from 24 to 48 h.p.f. cDNA using the following primers^36^: *apln* forward 5′-CACACACGCACACCACTACA-3′ and *apln* reverse 5′-CCATACTGGGCTTGAGCATT-3′; *apela* forward 5′-AACTGAACATTCCCACTCATCA-3′ and *apela* reverse 5′-TGAAAAGACAATACACTTTTAA-3′; *aplnra* forward 5′-CCGTGCTGTACATGCTCATT-3′ and *aplnra* reverse 5′-AGCGACTGCACCTCTGACTT-3′; *aplnrb* forward 5′-CCAGCTCCCTTTCTTCACAG-3′ and *aplnrb* reverse 5′-TCATCAGAGTTGGCTTGCAC-3′; *vegfr3* forward 5′-ATGAAGAGAGATTTTACGTTTTT-3′ and *vegfr3* reverse 5′-TTCCAGAGTATTAGCCCAGCAGG-3′; *klf2a* forward 5′-TTCCAGAGTATTAGCCCAGCAGG-3′ and *klf2a* reverse 5′-CTACATATGACGTTTCATATGA-3′. The T7 or SP6 promoter were added to the 5′-end of the reverse primer in a second round of amplification. T7-*apln* reverse 5′-GTAATACGACTCACTATAGGCCATACTGGGCTTGAGCATT-3′, T7-*apela* reverse 5′-GTAATACGACTCACTATAGGTGAAAAGACAATACACTTTTAA-3′, T7-*aplnra* reverse 5′-GTAATACGACTCACTATAGGAGCGACTGCACCTCTGACTT-3′, T7-*aplnrb* reverse 5′-GTAATACGACTCACTATAGGTCATCAGAGTTGGCTTGCAC-3′, SP6 *vegfr3* reverse 5′-GATTTAGGTGACACTATAGTTCCAGAGTATTAGCCCAGCAGG-3′ and SP6 *klf2a* reverse 5′-GATTTAGGTGACACTATAGCTACATATGACGTTTCATATGA-3′

### Assessment of blood vessel diameter and mean blood flow velocity

To assess blood vessel diameter, we imaged the trunk region of 84 h.p.f. *TgBAC(etv2:EGFP)* wild type, *klf2a*^*−/−*^ and *klf2a*^*−/−*^;*klf2b*^+/−^ larvae using a Zeiss LSM 800 confocal microscope. For quantitative analysis of blood vessel diameter in these animals, we analysed the mean blood vessel diameter of the DA and PCV from three different regions in the fifth and sixth somites.

To assess blood flow velocity, we performed time-lapse imaging of the trunk region of 84 h.p.f. wild type, *klf2a*^*−/−*^ and *klf2a*^*−/−*^;*klf2b*^+/−^ larvae using a Zeiss Spinning disc CSU-X1 confocal microscope with a high-speed camera and calculated blood flow velocity in the DA by measuring the time needed by RBCs to move 100 μm at the level of the fifth and sixth somites.

### Statistical analysis

Statistical significance for paired samples and for multiple comparisons was determined by Student's *t*-test and one-way analysis of variance with Tukey's test, respectively. Data were considered statistically significant if the *P*-value <0.05 (***).

### Data availability

The authors declare that data supporting the findings of this study are available within the article and its Supplementary Information files.

## Additional information

**How to cite this article:** Kwon, H.-B. *et al*. *In vivo* modulation of endothelial polarization by Apelin receptor signalling. *Nat. Commun.* 7:11805 doi: 10.1038/ncomms11805 (2016).

## Supplementary Material

Supplementary Information Supplementary Figures 1 - 9

Supplementary Movie 1 Endothelial cell polarization during migration. Time-lapse movie of a *Tg(kdrl:NLS-EGFP);Tg(fli1a:B4GALT1-mCherry)* embryo starting at 36 hpf.

Supplementary Movie 2 Endothelial cell polarization by blood flow. Time-lapse movie of a *Tg(kdrl:NLS-EGFP);Tg(fli1a:B4GALT1-mCherry)* embryo starting at 30 hpf.

Supplementary Movie 3 Endothelial cell polarization after cell division. Time-lapse movie of a *Tg(kdrl:NLS-EGFP);Tg(fli1a:B4GALT1-mCherry)* embryo starting at 30 hpf.

Supplementary Movie 4 Endothelial cell polarization is dependent on blood flow. Time-lapse movie of a *Tg(kdrl:NLS-EGFP);Tg(fli1a:B4GALT1-mCherry)* larva treated with 10 mM BDM starting at 96 hpf.
