# Supplementary material for: In vivo modulation of endothelial polarization by Apelin receptor signalling
Source: Nat Commun. 2016 Jun 1;7:11805. doi: 10.1038/ncomms11805 (PMC4895482; doi:10.1038/ncomms11805)
Supplement: Supplementary Information — Supplementary Figures 1 - 9 [file ncomms11805-s1.pdf]

Supplementary Figures

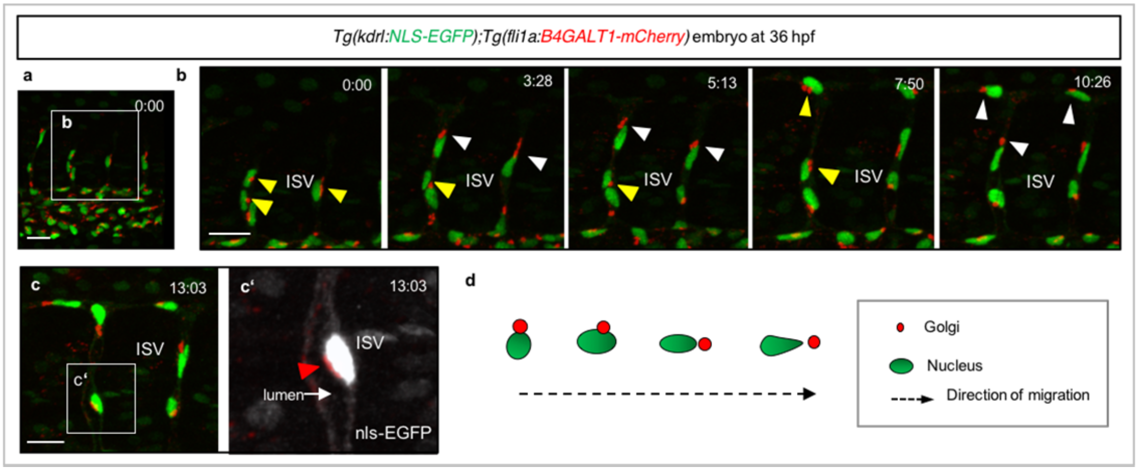

Supplementary Figure 1. Endothelial cell polarization during migration.

(a-c') 3D-rendered confocal stack time-lapse images of the trunk region of a 36 hpf *Tg(kdrl:NLS-EGFP);Tg(fli1a:B4GALT1-mCherry)* embryo. (b) Time-lapse images of the white box in (a). Time (hours:mins) is shown in the top right corner of the images. White arrowheads point to polarized ECs, yellow arrowheads to non-polarized ECs. (c) A confocal stack image of the white box in (a) at t=13:03. (c') Enlarged white box from (c). White arrow points to the lumen of the vessel. Red arrowhead points to the localization of the Golgi apparatus on the luminal side of the blood vessel. (d) Schematic representation of endothelial polarization during migration. Scale bars, 15 µm (a-c). Anterior to the left, dorsal to the top. DA, dorsal aorta; ISV, intersegmental vessel.

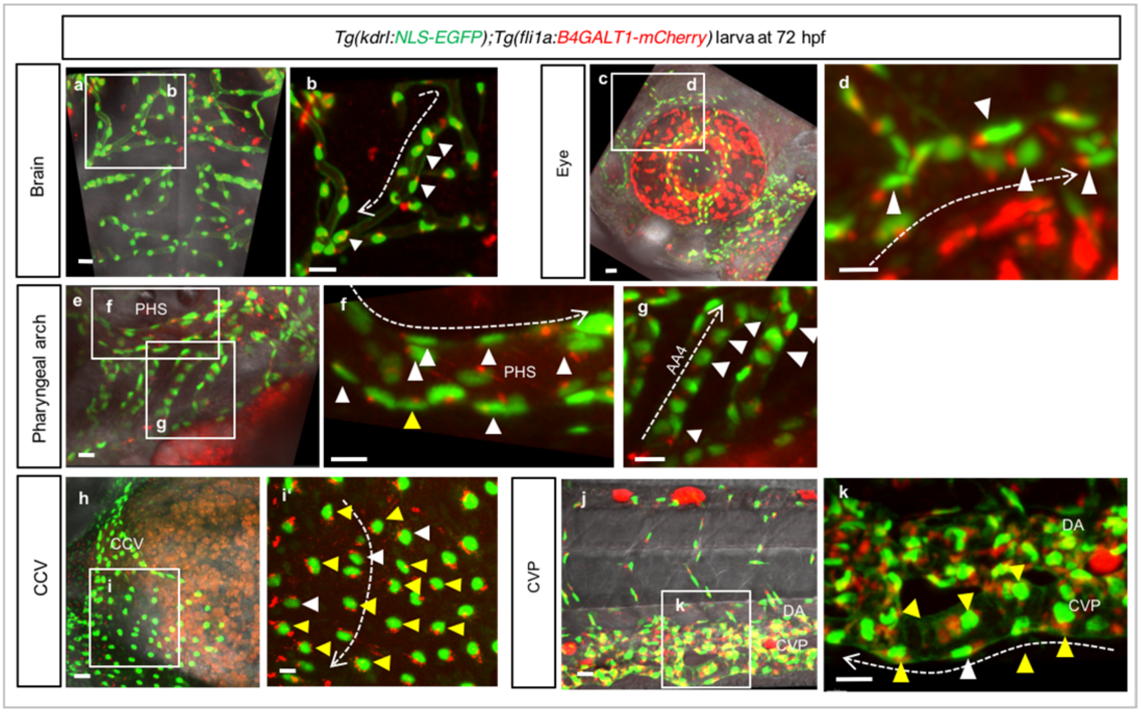

Supplementary Figure 2. Endothelial cell polarization in different vascular beds.

(a-k) 3D-rendered confocal stack images of different vascular beds: brain (a, b), eye (c, d), pharyngeal arch (e-g), CCV (h, i) and CVP (j, k) of a 72 hpf *Tg(kdrl:NLS-EGFP);Tg(fli1a:B4GALT1-mCherry)* larva. White arrowheads point to polarized ECs, yellow arrowheads to non-polarized ECs. Scale bars, 15 µm. White dashed arrows indicate the direction of blood flow. Anterior to the left, dorsal to the top. AA4, secondary pharyngeal arch; CCV, common cardinal vein; CVP, caudal vein plexus; DA, dorsal aorta; PHS, primary head sinus.

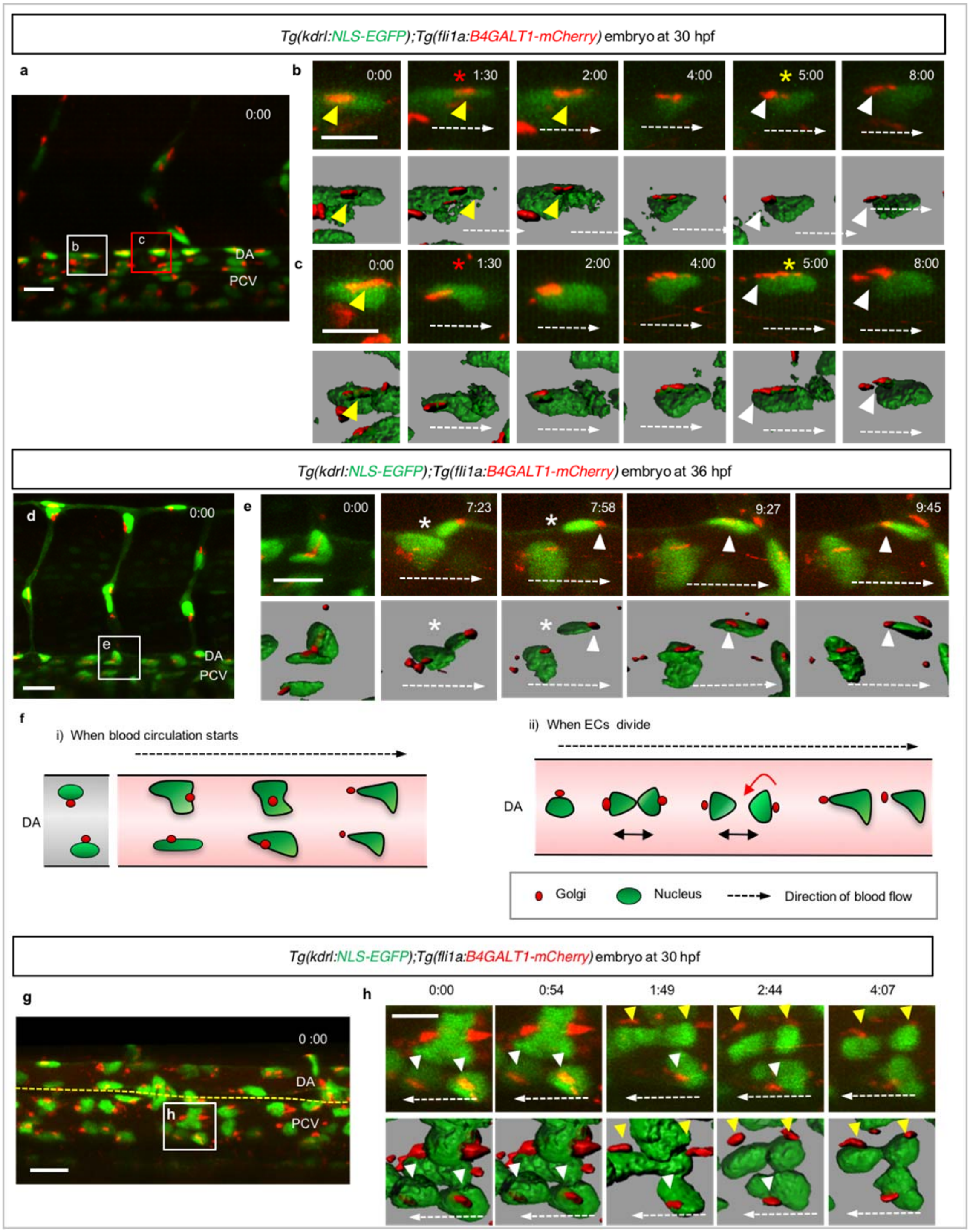

Supplementary Figure 3. Endothelial cell polarization in DA and PCV.

(a) 3D-rendered confocal stack images of the trunk region of a 30 hpf *Tg(kdrl:NLS-EGFP);Tg(fli1a:B4GALT1-mCherry)* embryo. (b, c) Confocal time-lapse images of the ECs in the white (b) and red (c) boxes in (a). Surface rendered images are displayed below. White arrowheads

point to polarized ECs, yellow arrowheads to non-polarized ECs. Red asterisk indicates the time when vigorous blood circulation starts. Yellow asterisk indicates the time when ECs are polarized. White dashed arrows indicate the direction of blood flow. (d) 3D-rendered confocal stack images of ECs undergoing cell division in the trunk region of a 36 hpf *Tg(kdrl:NLS-EGFP);Tg(fli1a:B4GALT1-mCherry)* embryo. (e) Time-lapse image of white box in (d). Surface rendered images are displayed below. White asterisks indicate ECs under cell division. White arrowheads point to the Golgi apparatus. White arrows indicate the direction of blood flow. Time (hours:mins) is shown in the top right corner of the images. (f) Schematic representations showing EC polarization after the onset of blood flow (i) and after cell division under vigorous blood flow (ii). (g, h) 3D-rendered confocal stack images of the trunk region of a 36 hpf *Tg(kdrl:NLS-EGFP);Tg(fli1a:B4GALT1-mCherry)* embryo. (h) Time-lapse images of the white box in (g). Surface rendered images are displayed below. White arrowheads and yellow arrowheads point to ECs undergoing cell division. Time (hours:mins) is shown in the top right corner of the images. Anterior to the left, dorsal to the top. Scale bars, 20 μm (a,d and g), 7 μm (b, c, e and h). DA, dorsal aorta; PCV, posterior cardinal vein.

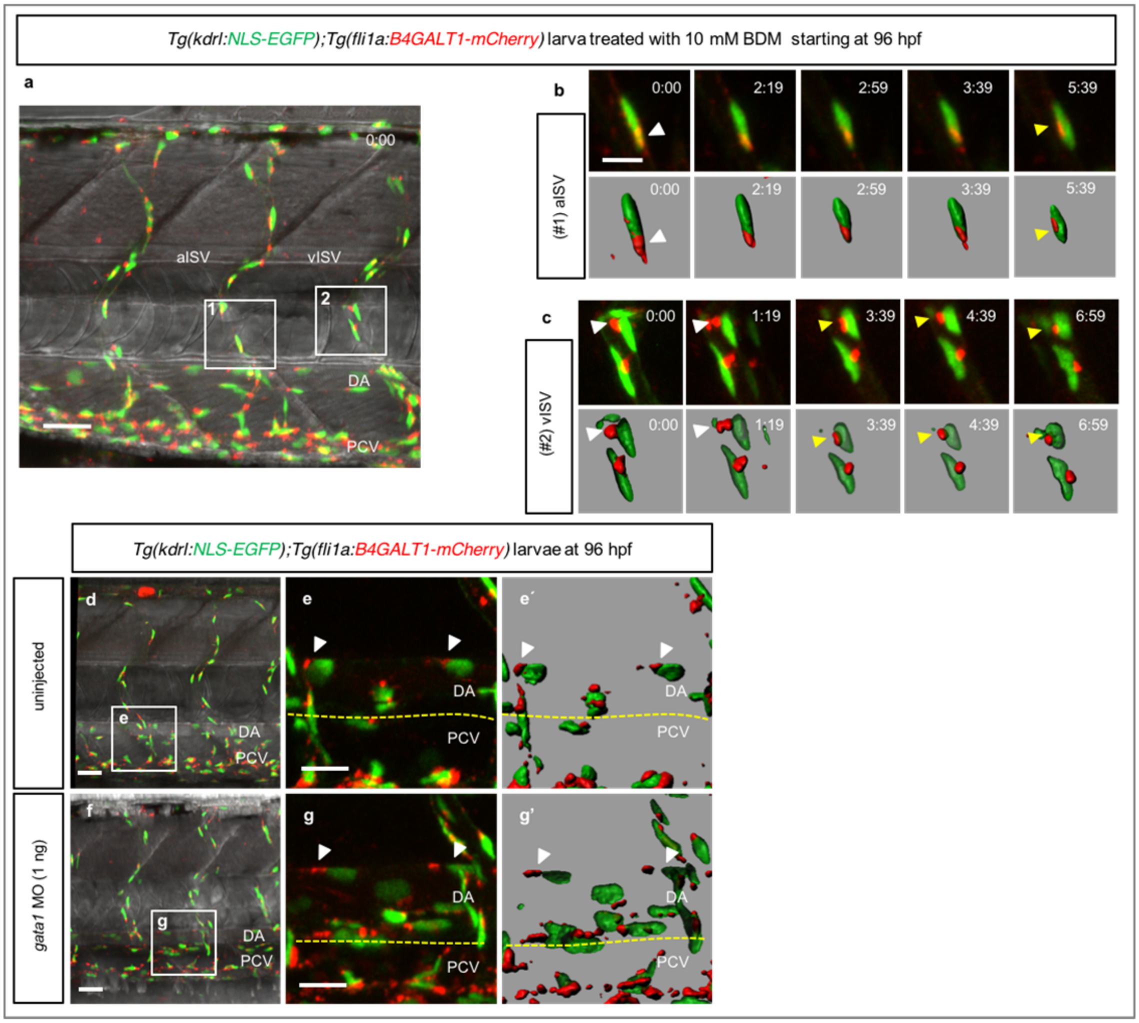

Supplementary Figure 4. Endothelial cell polarization is dependent on blood flow.

(a-c) 3D-rendered confocal stack images of the trunk region of a 48 hpf *Tg(kdrl:NLS-EGFP);Tg(fli1a:B4GALT1-mCherry)* embryo. (b, c) Time-lapse image of ECs identified in (a) (#1, b; #2, c). Surface-rendered images are displayed below. White arrowheads point to polarized ECs, yellow arrowheads to non-polarized ECs. Time (hours:mins) is shown in the top right corner of the images. (d, e, f and g) 3D-rendered confocal stack images of the trunk region of 96 hpf *Tg(kdrl:NLS-EGFP);Tg(fli1a:B4GALT1-mCherry)* uninjected (d, e) and *gata1* morphant (f, g) larvae. The white boxes in the left panels (d, f) are enlarged in the middle panels (e, g). Surface-rendered images of boxed areas are shown in the right panels (e’, g’). White arrowheads point to polarized ECs. Anterior to the left, dorsal to the top. Scale bars, 20 μm (a, d, e,f and g), 7 μm (b, c). DA, dorsal aorta; PCV, posterior cardinal vein; ISV, intersegmental vessel.

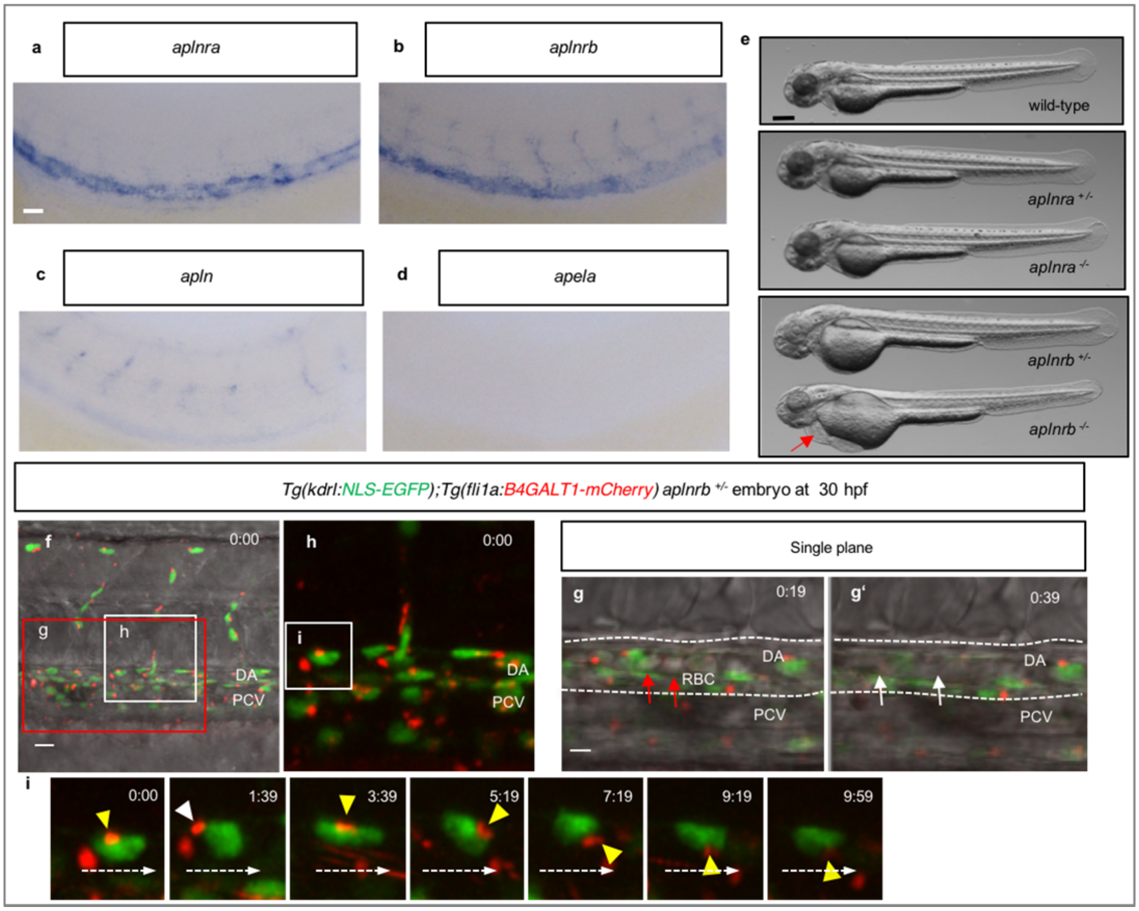

Supplementary Figure 5. *aplnrb* modulates endothelial cell polarization.

(a-d) *in situ* hybridization of 48 hpf wild-type embryos for *aplnra* (a), *aplnrb* (b), *apln* (c) and *apela* (d) expression. (e) Bright field images of 60 hpf wild-type, *aplnra* $^{+/-}$, *aplnra* $^{-/-}$, *aplnrb* $^{+/-}$ and *aplnrb* $^{-/-}$ embryos. (f-g') 3D-rendered confocal stack images of a 72 hpf *Tg(kdrl:NLS-EGFP);Tg(fli1a:B4GALT1-mCherry) aplnrb* $^{+/-}$ larva. The white box in (f) is enlarged in (h) and the single plane images of the red box in (f) are enlarged in the right panels (g, g'). (g, g') At t=0:19, red blood cells (RBCs, red arrows) in the DA are mainly stationary whereas by t=0:39 (39 mins) they are moving too fast to be clearly distinct (white arrows). (i) Time-lapse confocal images of DA ECs in the white box in (h). White dashed arrows indicate the direction of blood flow. White arrowheads point to polarized ECs, yellow arrowheads to non-polarized ECs. Time (hours:mins) is shown in the top right corner of the images. Anterior to the left, dorsal to the top. Scale bars, 40 µm (a-d), 100 µm (e), 20 µm (f-i). DA, dorsal aorta; PCV, posterior cardinal vein.

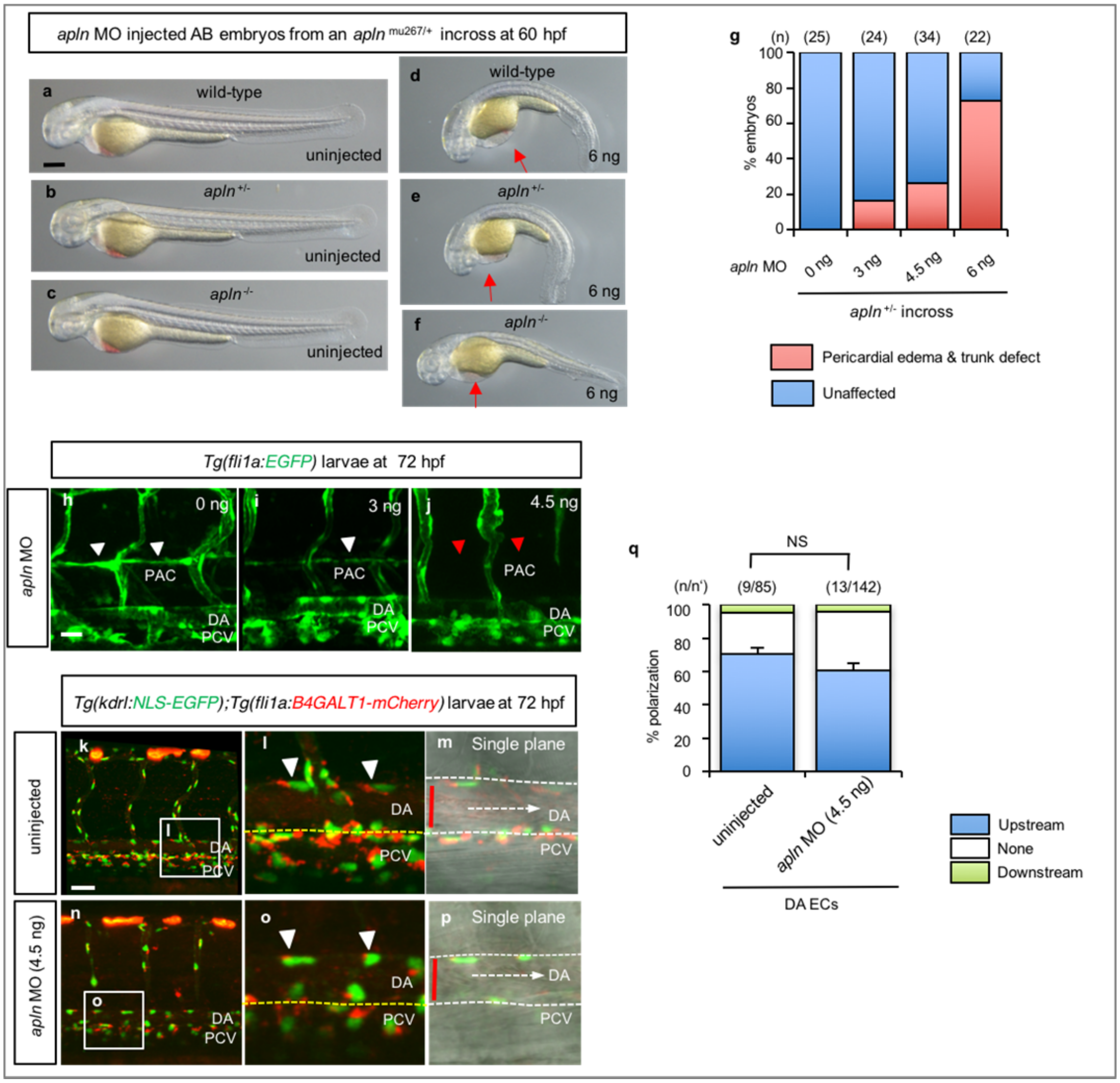

Supplementary Figure 6. Endothelial cell polarization appears unaffected in *apln* morphants. (a-f) Bright field images of 60 hpf embryos from an *apln*$^{+/-}$ incross uninjected (a-c) or injected with *apln* MO (6 ng) (d, e, f). Red arrows point to pericardial edema. (g) Quantative analysis of 60 hpf *apln* MO injected embryos from an *apln*$^{+/-}$ incross. The numbers of larvae (n) are indicated above the graph. (h-j) 3D rendered confocal images of 72 hpf *Tg(fli1a:EGFP)* larvae injected with *apln* MO (4.5 ng). White arrowheads point to the PAC vessel (h, i). Red arrowheads indicate absence of PAC in *apln* morphants (j). (k-p) Confocal images (lateral views) of 72 hpf *Tg(kdrl:NLS-EGFP);Tg(fli1a:B4GALT1-mCherry)* larvae uninjected (k-m) or injected (n-p) with *apln* MO (4.5 ng). The white boxes in the left panels (k, n) are enlarged in the middle panels (l, o) and the single plane images, with brightfield, of those boxed areas are enlarged in (m) and (p). (q) Quantitative

analysis of EC polarization in 72 hpf uninjected and *apln* MO injected (4.5 ng) larvae. The numbers of larvae (n) and ECs (n') are indicated above the graph. Anterior to the left and dorsal to the top. Scale bars, 100 µm (a-f), 20 µm (h-p). DA, dorsal aorta; PCV, posterior cardinal vein. Error bars, SEM.

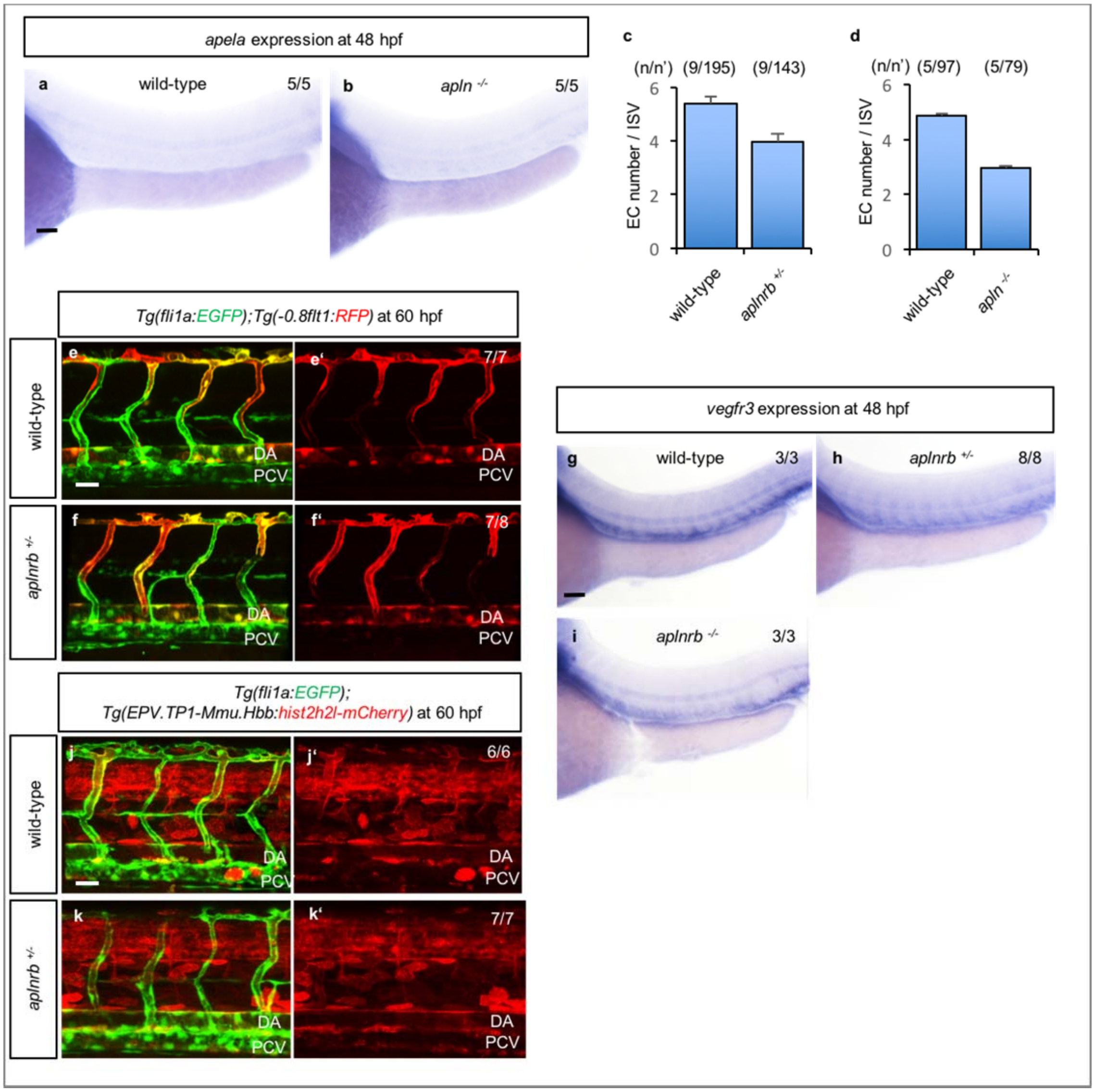

Supplementary Figure 7. ***apln*** **mutants show reduced endothelial cell numbers.**

(a-b) *in situ* hybridization of 48 hpf wild-type (a) and *apln* $^{-/-}$ (b) embryos for *apela* expression. The numbers of embryos examined are indicated in the top right corner of the images. (c, d) Quantitative analysis of EC numbers per three somites in 72 hpf *aplnrb*$^{+/-}$ (c) and *apln* $^{-/-}$ (d) larvae. The numbers of larvae (n) and ECs (n') are indicated above the graph. (e-f') Confocal images (lateral views) of 60 hpf *Tg(fli1a:EGFP); Tg(-0.8flt1:RFP)* wild-type (e, e') and *aplnrb*$^{+/-}$ (f, f') embryos. (g-i) *in situ* hybridization of 48 hpf wild-type (g), *aplnrb* $^{+/-}$ (h) and *aplnrb* $^{-/-}$ (i) embryos for *vegfr3* expression. (j-k') Confocal images (lateral views) of 60 hpf *Tg(fli1a:*EGFP*); Tg(EPV.TP1-Mmu.Hbb:hist2h2l-mCherry)* wild-type (j, j') and *aplnrb*$^{+/-}$ (k, k') embryos. The numbers of embryos examined are in the

top right corner of the images (e’, f’, g-i, j’, k’). Anterior to the left, dorsal to the top. Scale bars, 60 μm (a-b, g-i) , 20 μm (e-f’, j-k’). DA, dorsal aorta; PCV, posterior cardinal vein. Error bars, SEM.

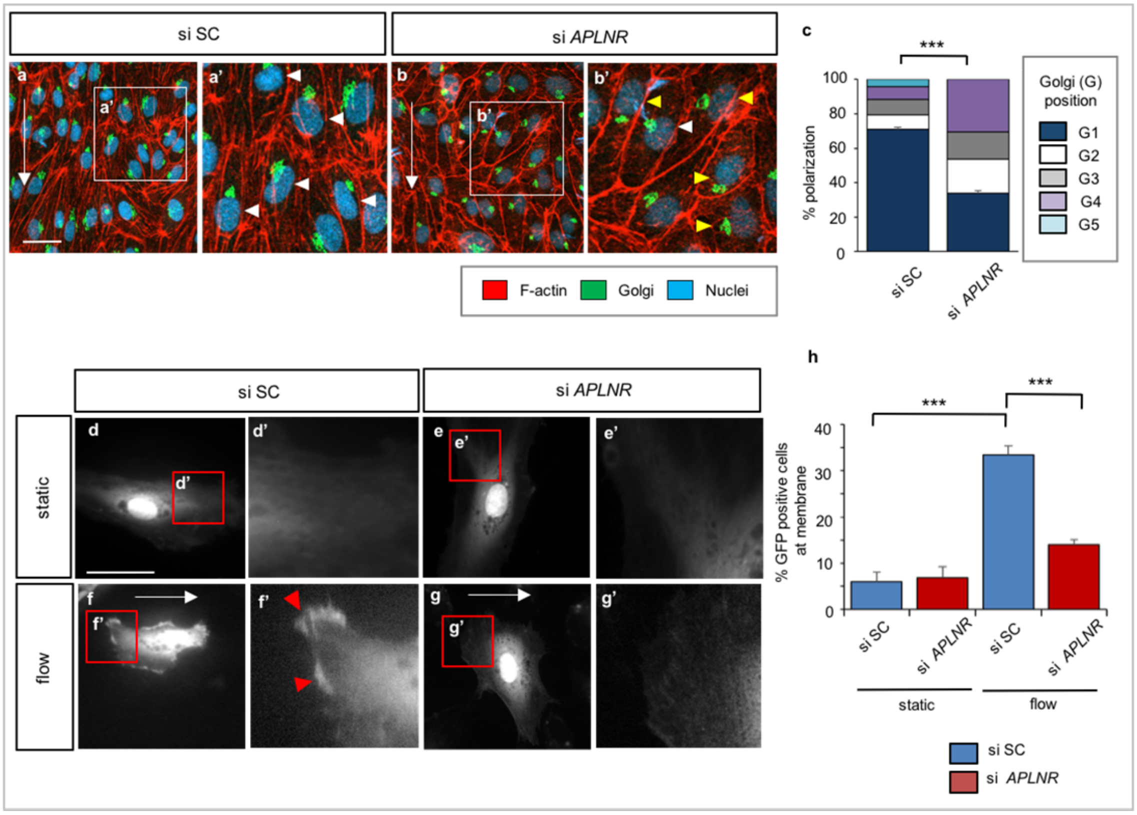

Supplementary Figure 8. APLNR signaling modulates the polarization of human arterial endothelial cells.

(a-b') Immunofluorescence staining of HUAECs transfected with si SC or si *APLNR* subjected to laminar flow at 20 dynes/cm^2 for 18 hours. Cells were fixed and stained with the GM130 Golgi antibody (green), Phalloidin (red) and DAPI (blue). White arrowheads point to polarized ECs (i.e., Golgi apparatus positioned within ±45° against the direction of flow (G1)). Yellow arrowheads point to depolarized ECs (i.e., Golgi positioned within + 45° to +180° and +180° to -45° against the direction of flow (G2-G4)). (c) Quantification of polarization of HUAECs subjected to laminar flow at 20 dynes/cm^2 for 18 hours transfected with si SC or si *APLNR*. n > 300 cells, from at least three independent experiments. (d-g') Fluorescent images of HUAECs transfected with ARRB-GFP as well as si SC or si *APLNR* subjected to laminar flow at 20 dynes/com^2 for 15 mins. Red arrowheads point to plasma membrane localization of ARRB-GFP after 15 mins of laminar flow. (h) Quantification of ARRB-GFP membrane localization of HUAECs subjected to static condition or

laminar flow at 20 dynes/cm^2 for 15 mins transfected with si SC or si *APLNR*. *** $P<0.05$. Scale bars, 20 μm. Error bars, SEM.

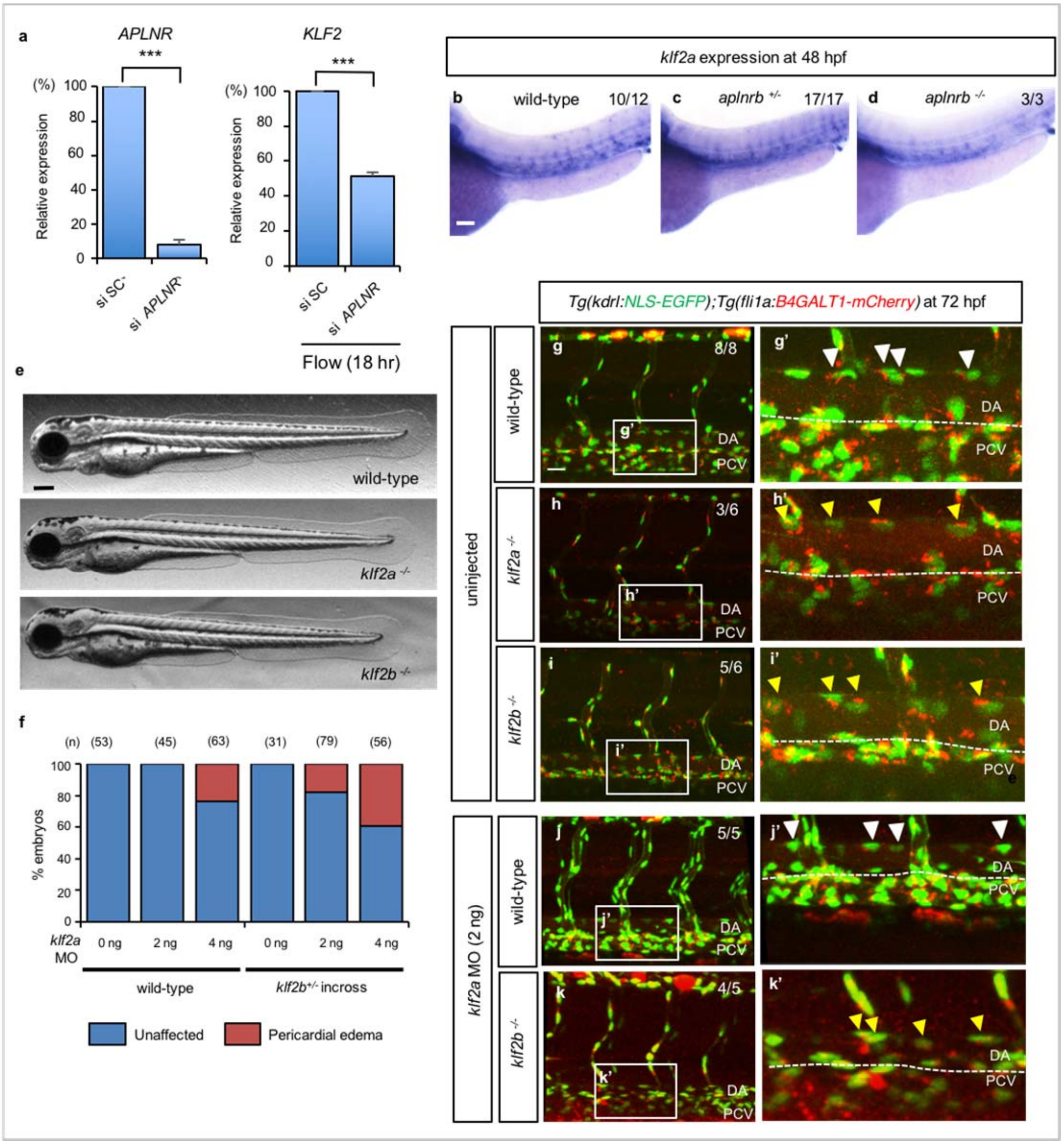
a
APLNR
KLF2
***
(%)
Relative expression
si SC
si APLNR
Flow (18 hr)
klf2a expression at 48 hpf
b wild-type 10/12
c aplnrb +/- 17/17
d aplnrb -/- 3/3
Tg(kdrl:NLS-EGFP);Tg(fli1a:B4GALT1-mCherry) at 72 hpf
uninjected
klf2a MO (2 ng)
wild-type
klf2a -/-
klf2b -/-
g 8/8
h 3/6
i 5/6
j 5/5
k 4/5
DA
PCV
e
f
% embryos
(n) (53) (45) (63) (31) (79) (56)
klf2a MO
0 ng 2 ng 4 ng 0 ng 2 ng 4 ng
wild-type
klf2b+/- incross
Unaffected
Pericardial edema

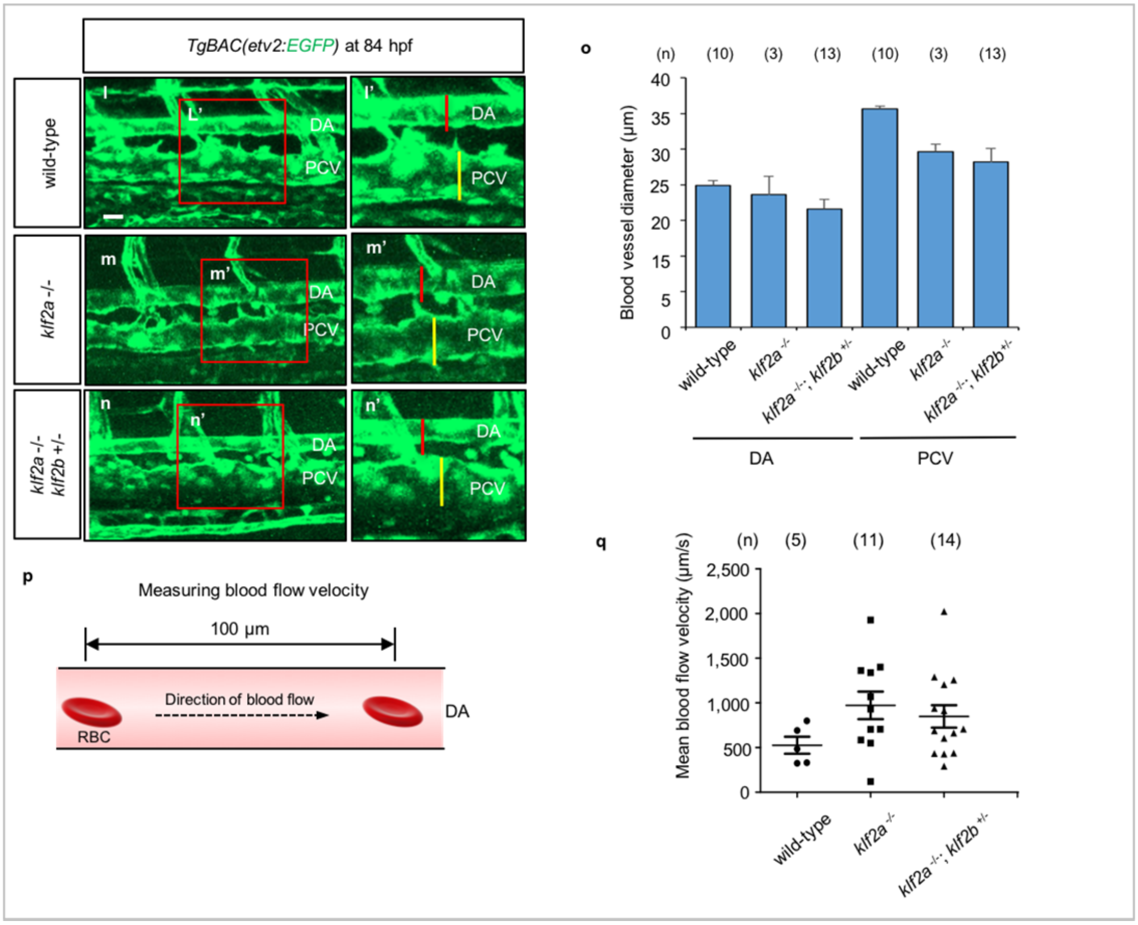

Supplementary Figure 9. *KLF2* regulation by APLNR and endothelial cell polarization in *klf2* mutants.

(a) qPCR analysis. Validation of efficacy of siRNA for *APLNR* in HUVECs (left). *KLF2* expression in *APLNR* KD HUVECs transfected with si SC or si *APLNR* subjected to laminar flow at 12 dynes/cm^2 for 18 hours (right). *** P<0.05. *KLF2* expression is downregulated in HUVECs after *APLNR* knock-down. (b-d) *in situ* hybridization for *klf2a* expression in 48 hpf wild-type (b), *aplnrb*$^{+/-}$ (c) and *aplnrb* $^{-/-}$(d) embryos. The numbers of embryos examined are indicated in the top right corner of the images. (e) Brightfield images of 72 hpf wild-type, *klf2a* $^{-/-}$ and *klf2b* $^{-/-}$ *larvae*. (f) Quantification of larvae showing pericardial edema at 72 hpf. *klf2a* MO (2 and 4 ng) was injected into one-cell stage embryos from wild-type or *klf2b*$^{+/-}$ incrosses. (f-k') Confocal images of 72 hpf *Tg(kdrl:NLS-EGFP);Tg(fli1a:B4GALT1-mCherry)* wild-type (g, g'), *klf2a* $^{-/-}$ (h, h'), *klf2b*$^{-/-}$ (i, i'), wild-type injected with *klf2a* MO (2ng) (j, j') and *klf2b*$^{-/-}$ injected with *klf2a* MO (2ng) (k, k') larvae. The white boxes in the left panels (g, h, i, j and k) are enlarged in the right panels (g', h', i', j' and k').

White dashed lines denote the ventral boundary of the DA. White arrowheads point to polarized ECs, yellow arrowheads to non-polarized ECs. The numbers of larvae examined are indicated in the top right corner of the images (g, h, i, j, k). (l-o’) Confocal images of 84 hpf *TgBAC(etv2:EGFP)* wild-type (l, l’), *klf2a*$^{-/-}$ (m, m’) and *klf2a*$^{-/-}$*; klf2b*$^{+/-}$ (n, n’) larvae. The red boxes in the left panels (l, m and n) are enlarged in the right panels (l’, m’ and n’). Red bars represent the diameter of the wild-type DA, yellow bars the diameter of the wild-type PCV. (o) Quantification of blood vessel diameter in 84 hpf wild-type, *klf2a*$^{-/-}$ and *klf2a*$^{-/-}$*; klf2b*$^{+/-}$ larvae. The numbers of larvae examined are indicated in the top of the graph. (p) Schematic representation of blood flow velocity measurements. (q) Quantification of blood flow velocity in 84 hpf wild-type, *klf2a*$^{-/-}$ and *klf2a*$^{-/-}$*; klf2b*$^{+/-}$ larvae. The numbers of larvae examined are indicated in the top of the graph. Anterior to the left, dorsal to the top. Scale bars, 60 µm (b-d), 100 µm (e), 20 µm (g-n). DA, dorsal aorta; PCV, posterior cardinal vein. Error bars, SEM.
